# Psychosocial factors influencing shared bicycle travel choices among Chinese: An application of theory planned behavior

**DOI:** 10.1371/journal.pone.0210964

**Published:** 2019-01-25

**Authors:** Zhang Xin, Ma Liang, Wang Zhanyou, Xing Hua

**Affiliations:** 1 School of Management Science and Engineering, Shandong University of Finance and Economics, Jinan, China; 2 Office of Academic Research, Shandong Management University, Jinan, China; 3 Computer Science and Technology, Shandong Technology and Business University, Yantai, China; Qazvin University of Medical Sciences, ISLAMIC REPUBLIC OF IRAN

## Abstract

The worldwide rise of shared bicycle use has changed the way people travel. Here we analyze shared bicycle use from the perspective of the theory of planned behavior, and propose a model to investigate factors influencing shared bicycle usage in China. A total of 211 shared bicycle users selected from 28 provinces throughout China completed a self-reported survey. Structural equation modelling (SEM) was used to delineate the pathway from shared bicycle usage. The SEM model demonstrated that: (1) shared bicycle use intention was significantly associated with four variables, namely travel attitude(β = 0.491, t = 24.569), social norms(β = 0.149, t = 6.771), travel habits(β = 0.146, t = 7.226) and perceived behavioral control (β = 0.190, t = 11.110); (2) shared bicycle use behavior was significantly affected by shared bicycle use intention(β = 0.406, t = 15.936), and also by travel habits(β = 0.320, t = 11.921); (3) shared bicycle use behavior was also affected by demographic variables (gender, age) and situational factors (distance). The conclusions of this study provide useful data for operators of bicycle services and government policy makers.

## Introduction

In recent years, smog has become a serious problem faced by city dwellers in China [1]. As a result, citizens have become increasingly aware of the role of individual behavior in improving environmental conditions [2]. Since 2016 shared bicycle services have significantly expanded in China[3]. The service is provided by bicycle operators on campuses, in subway stations, bus stations, residential areas and business districts on a time-sharing lease mode. Due to its low-carbon concept, popular support of shared bicycle use has dramatically increased in China[4]. As of June 2017, shared bicycle users amounted to 106 million [5]. In cities with heavy traffic and under environmental stress due to pollution, shared bicycle services provide a simple, economical and efficient travel solution[6].

However, multiple factors may influence the decision-making process underlying travel selection [7, 8]. Although residents may be sympathetic to pollution-free travel, many potential users give more importance to travel distance [9], weather conditions [10], convenience [11] and personal values [12]. Based on the theory of planned behavior [13–15], some studies proposed that factors such as environmental awareness, attitude, perceived behavioral control, subjective norms and guiding language can positively affect travel intentions and actual behavior[16, 17]. To date, few studies have specifically addressed the factors underlying the decision-making process behind shared bicycle use. Compared with cars or motorbikes, shared bicycle use presents various unique features. First, shared bicycle stations should be near prospective users, which may be an issue particularly in smaller towns. Second, it requires a downloadable shared bicycle app and payment of a deposit, which may be a problem for individuals unfamiliar with smart phones (a more likely circumstance among older individuals). Third, long-distance riding may be less comfortable and energy-costly than driving a car or motorbike. Fourth, a shared bicycle can be parked in an available bicycle station, while a car of motorbike requires a parking lot. Finally, shared bicycle travel involves no pollutant emissions. Therefore, some factors seem to promote, and others prevent shared bicycle use, but their relative strength remains largely unexplored. Furthermore, demographic (gender, age) and situational factors such as distance may also affect the choice of shared bicycle travel and have not been thoroughly investigated.

To fill these gaps, we present a study of shared bicycle use intention in China, based on the theory of planned behavior and on data from an online survey of factors affecting the choice to use shared bicycles. Our study of determinants of shared bicycle use behavior in China may help operators of shared bicycle services to more effectively carry out their marketing and planning activities.

## Theoretical background

### Theory of planned behavior

The theory of planned behavior (TPB) is one of the most important models of the relationship between attitude and behavior in the field of social psychology. The theory was derived from the theory of reasoned action [18], which postulates that subjective norms and behavioral attitudes determine individual behavioral intentions, and the latter determines individual behavior. Furthermore, individual behavioral intentions are also affected by individual capacity, or ‘perception behavioral controls’ [19]. The inclusion of perceived behavioral control led to the formalization of the theory of planned behavior [20]. In theory, subjective norms mainly refer to the influence of important people or organizations on individual behavior. Behavioral attitude refers to an individual's positive or negative feelings towards the behavior, and perceived behavioral control refers to the degree of difficulty associated with performance of the behavior [21].

The theory of planned behavior has been widely applied in the field of social psychology and information systems[22]. It is recognized as a powerful model for predicting behavioral intentions[23], and was recently extended to the field of transportation [24]. According to the theory, individual behavior is positively influenced by individual behavior intention, which in turn is positively influenced by subjective norms, perceived behavioral control and behavior attitudes. Here we apply TPB to investigate the factors underlying the decision to adopt shared bicycle travel in China. We propose four hypotheses:

H1: Perceived behavioral control positively affects shared bicycle use intention.

H2: Social norms positively affect shared bicycle use intention.

H3: Behavior attitude positively affects shared bicycle use intention.

H4: Shared bicycle use intention positively affects shared bicycle use behavior.

### The relationship between travel habits and travel intentions

Habits refer to regular, nonjudgmental, or unconscious behavior [25]. User habits have been widely studied in psychology [26] and information systems [27]. Behavioral habits were shown to predict future behavioral intentions and actual behavior [28, 29]. Behavioral habits play a major role in routine choice behavior, while behavioral intentions can play a leading role in behavioral choice in new environments[30]. Behavioral habits and intentions can simultaneously determine behavior [31], although strong behavioral habits can also affect actual behavior [21]. Since 2016, shared bicycle use has been a new traveling option and changed travel habits across the world [32]. Since the habit of using shared bicycle trips is likely to promote shared bicycle use intention and behavior, we also propose the additional hypotheses:

H5: Travel habits positively affect shared bicycle use intention.

H6: Travel habits positively affect shared bicycle use behavior.

**Fig 1:**
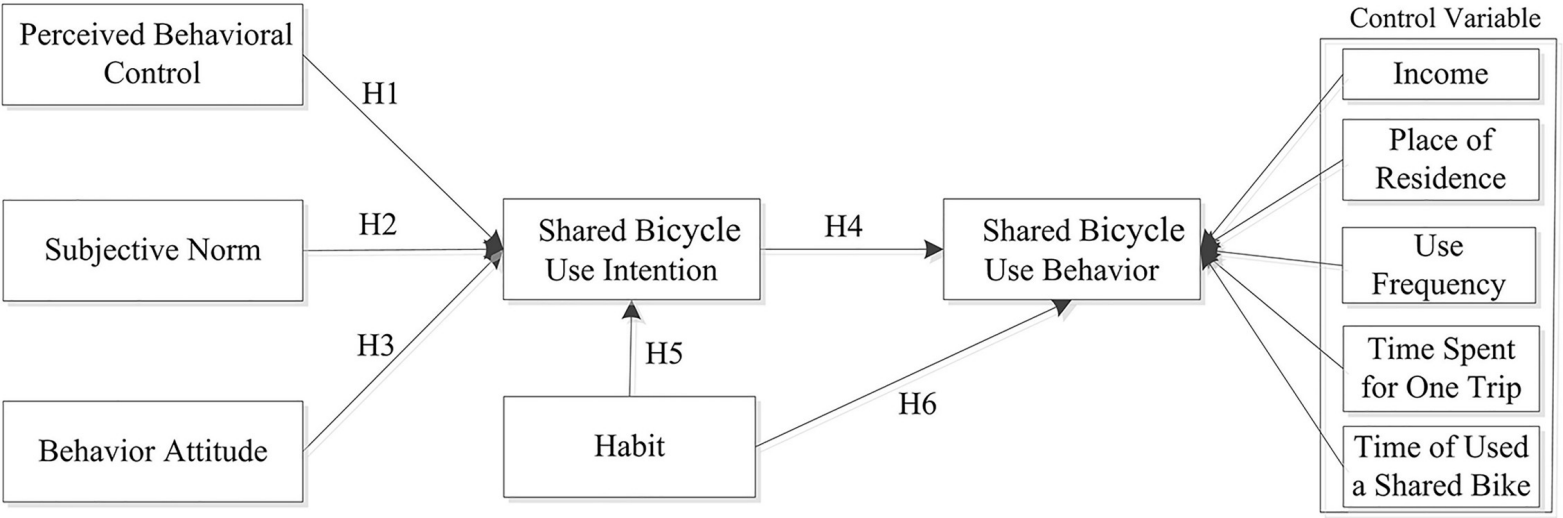
Psychosocial factors influencing shared bicycle travel choices: an application of theory planned behavior.

In addition, we tested the effects of the following control variables: user monthly income, residential area (urban or suburban), frequency of shared bicycle use, travel time spent per trip, and duration of shared bicycle use. Our research model and predictions are summarized in Fig 1.

## Materials and methods

### Variables and measures

A self-rated questionnaire was given to an online sample of Chinese respondents and used to validate the conceptual model. Levels of agreement to short statements taken from prior literature were used to quantify each variable (Table 1). Four short statements or measures of perceived behavioral control, three of subjective norm, and three of behavior attitude, were adapted from Azjen [20] and Donald et al. [33]. Three measures of behavior intention were derived from Chen [34]. Three measures of travel habit were adapted from Khalifa and Liu [35]. Shared bicycle use behavior was derived from Courtois et al. [36]. Agreement to each item was measured on a 7-point Likert scale ranging from 1 (strongly disagree/unlikely) to 7 (strongly agree/likely).

**Table 1:** Factors and corresponding measures.

Factor	Measure Items
Perceived Behavioral Control (PBC)	PBC1 It is easy to learn how to use a shared bicycle.
	PBC2 I believe I am capable of learning how to use a shared bicycle.
	PBC3 It's easy for me to use a shared bicycle.
	PBC4 For me, it's simple to use a shared bicycle.
Subjective Norm (SN)	SN1 The people who are most important to me support my use of shared bicycles.
	SN2 The people who are most important to me think I should use a shared bicycle.
	SN3 The government gives priority to the development of shared bicycle transportation policy, and I support the use of shared bicycle.
Behavior Attitude (AT)	AT1 It makes sense to use a shared bicycle for travel.
	AT2 It is valuable to me to use a shared bicycle for travel.
	AT3 The use of shared bicycle for travel is a wise act.
Shared Bicycle Use Intention (BI)	BI1 I want to use a shared bicycle for travel.
	BI2 I'm going to use a shared bicycle for travel.
	BI3 I would like to use a shared bicycle for travel.
Habit (HA)	HA1 Shared bicycle has become my natural choice of travel at short distances.
	HA2 When I travel at short distances, use of a shared bicycle comes to my mind
	HA3 Shared bicycle has become a spontaneous short distance travel option to me.
Shared bicycle use behavior (AB)	AB1 I've used a shared bicycle.
	AB2 In the past I have used a shared bicycle.
	AB3 For a short trip, I have used a shared bicycle.

### Data collection

The surveys used in this study were distributed by a Chinese website (Soujump.com) providing online survey services, and this platform has been used in numerous previous studies for distributing questionnaires[37, 38]. To ensure data quality, this study used the platform's paid sample service, which provides more than 2.6 million sampling resources from different cities in China with diverse demographic backgrounds. The platform sends email invitations to its registered members inviting them to complete a questionnaire. If members respond to the invitation and complete the survey, the platform charges the client 1–96 Chinese yuan per response, depending on the complexity of the surveys [39]. We employed the platform to randomly select 315 members from their pool of registered members and then to send email invitations to them to complete our questionnaire. A total of 315 questionnaires were received from 28 provinces throughout the country. We eliminated subjects providing the same answer to all questions, those with no experience with shared bicycle usage, and those who finished the survey in less than five minutes. The final sample consisted of 211 questionnaires (a questionnaire efficiency of 67%). In order to determine whether the sample size was sufficient, we used Soper’s *a priori* sample size calculator for structural equation models[40]. The results showed that our sample size was much larger than the minimum required sample size 95, meaning that it was more than sufficient. The protocol and informed consent forms for this study were approved by the Ethics Committee in Research of the Institute of Management Science of Shandong University of Finance and Economics. The demographic information included gender, age, education, income and occupation (Table 2).

**Table 2:** Descriptive statistics of respondent characteristics.

Demographic variable		Size	%
Gender	Male	99	46.92
	Female	112	53.08
Age	< = 20 years old	1	0.47
	21–30 years old	83	39.34
	31–40 years old	88	41.71
	40–50 years old	31	14.69
	>50 years old	8	3.79
Personal Income (RMB)	< = 3,000	16	7.58
	3,001–5,000	58	27.49
	5,001–8,000	83	39.34
	> 8,001	54	25.59
Occupation	A government office/institution, etc.	21	9.95
	Enterprise staff	161	76.30
	Individual occupation	13	6.16
	Student	11	5.21
	Others	5	2.37

Shared bicycle use was assessed by the results of the questionnaires (Table 3). The majority of respondents reported that their first choice of shared bicycle brand was OFO, followed by Mobike. Most respondents said that they often used a shared bicycle. The bicycling distance was generally less than three kilometers, indicating that most respondents used a bicycle primarily when traveling a short distance. Most of the respondents had been using shared bicycles for three to six months, followed by the second largest group who had used the service six to twelve months. Most respondents’ said they preferred to spend from eleven to thirty minutes per shared bicycle trip, followed by the next largest group at thirty-one to sixty minutes. Nearly a third of respondents indicated a distance between work place and residence of one to three km, and most said they preferred using a shared bicycle for work, but also sometimes for entertainment and shopping purposes. More than half of the respondents owned a car, while close to half owned a storage battery car, and about a third owned a personal bicycle. Finally, the vast majority of the respondents said they lived in urban areas, while a tiny percentage of respondents said they lived in the suburbs.

**Table 3:** Usage of shared bicycles.

Items	Classifications	Size	%	Items	Classifications	Size	%
Brand of shared bicycle	OFO	143	67.77	Distance between work place and residence	1 kilometer and below	16	7.58
	Mobike	60	28.44		1–3 kilometers	59	27.96
	Others	8	3.79		3–5 kilometers	56	26.54
Use frequency of shared bicycle	Occasionally used (many times a month)	79	37.44		5–10 kilometers	54	25.59
	Often used (a number of times a week)	120	56.87		10–15 kilometers	19	9.00
	Frequency use (many times a day)	12	5.69		15 kilometers and above	13	6.16
Travel distance use shared bicycle	1 kilometer and below	22	10.43	Purpose of using shared bicycle	Go for work	74	35.07
	1–2 kilometer	77	36.49		Shopping	46	21.80
	2–3 kilometers	81	38.39		Go to school	8	3.79
	3–4 kilometers	21	9.95		Got home	9	4.27
	5 kilometers and above	10	4.74		Recreation & Entertainment	66	31.28
Time of used a shared bicycle	3 months and below	27	12.80		Others	8	3.79
	3–6 months	82	38.86	Other transportation vehicles at home	Car	144	68.25
	6–12 months	61	28.91		Bicycle	69	32.70
	1 years and above	41	19.43		A storage battery car	93	44.08
Time spent on shared bicycle for one trip	Less than 10 minutes	16	7.58		Motorcycle	29	13.74
	11–30 minutes	141	66.82		Others	20	9.48
	31–60 minutes	47	22.27	Place of residence	Urban areas	195	92.42
	More than 1 hours	7	3.32		Suburbs	16	7.58

### Measurement model

We first tested the reliability and validity of the model. According to Hair et al. [41], validity was established by Cronbach's alpha > 0.7, composite reliability > 0.7, and average variance extracted (AVE) > 0.5. Cronbach's coefficients of all measurement items were above 0.7, indicating that the internal consistency of measurement items was acceptable (Table 4). Composite reliability values of all constructs were above 0.8 and therefore acceptable. Scale validity was assessed by the convergence validity and discriminant validity of the structure. The AVE value of all measured items was above 0.6, indicating that the measurement model had good convergence validity (Table 4). The square root of AVE of measured variables was greater than their correlation coefficients, indicating that the scale had good discriminant validity. Finally, Table 5 shows the factor loadings and the cross factor loading of the measured items. All measurement variables and their potential variables displayed high correlation coefficients, while correlation coefficients of other latent variables were relatively low, indicating that the measured items had good distinction and internal consistency. Smart PLS2.0 was used in the study.

**Table 4:** Descriptive statistics and inter-construct correlations.

Items	Cronbach’s Alpha	Composite Reliability	AVE	AB	AT	PBC	BI	HA	SN
AB	0.744	0.853	0.660	0.812					
AT	0.803	0.884	0.718	0.539	0.847				
PBC	0.862	0.906	0.707	0.680	0.556	0.841			
BI	0.835	0.893	0.737	0.614	0.596	0.616	0.858		
HA	0.844	0.905	0.761	0.562	0.628	0.523	0.666	0.872	
SN	0.817	0.890	0.730	0.531	0.612	0.515	0.607	0.752	0.854

Note: Shared bicycle use behavior (AB); Behavior attitude (AT); Perceived behavioral control (PBC); Shared bicycle use intention (BI); Habit (HA); Social norms (SN).

**Table 5:** Cross loadings.

Items	AB	AT	PBC	BI	HA	SN
AB1	**0.831**	0.417	0.598	0.469	0.371	0.381
AB2	**0.816**	0.371	0.552	0.476	0.466	0.415
AB3	**0.789**	0.515	0.504	0.548	0.538	0.499
AT1	0.490	**0.859**	0.527	0.676	0.535	0.588
AT2	0.393	**0.841**	0.362	0.657	0.464	0.555
AT3	0.484	**0.841**	0.519	0.689	0.594	0.665
PBC1	0.548	0.482	**0.806**	0.484	0.466	0.472
PBC2	0.613	0.428	**0.856**	0.550	0.425	0.430
PBC3	0.582	0.503	**0.852**	0.531	0.428	0.412
PBC4	0.540	0.461	**0.849**	0.505	0.447	0.425
BI1	0.526	0.681	0.551	**0.859**	0.567	0.589
BI2	0.576	0.740	0.561	**0.933**	0.635	0.673
BI3	0.469	0.633	0.477	**0.777**	0.481	0.545
HA1	0.416	0.577	0.423	0.585	**0.860**	0.677
HA2	0.484	0.513	0.462	0.533	**0.870**	0.615
HA3	0.553	0.559	0.478	0.621	**0.887**	0.678
SN1	0.358	0.550	0.356	0.551	0.669	**0.865**
SN2	0.413	0.555	0.377	0.541	0.591	**0.863**
SN3	0.560	0.694	0.555	0.692	0.657	**0.834**

Note: Shared bicycle use behavior (AB); Behavior attitude (AT); Perceived behavioral control (PBC); Shared bicycle use intention (BI); Habit (HA); Social norms (SN). AB1 is the first measurement item of AB; AB2 is the second measurement item of AB; AB3 is the third measurement item of AB. Other measurement items are similar.

## Results

### Structural model

**Fig 2:**
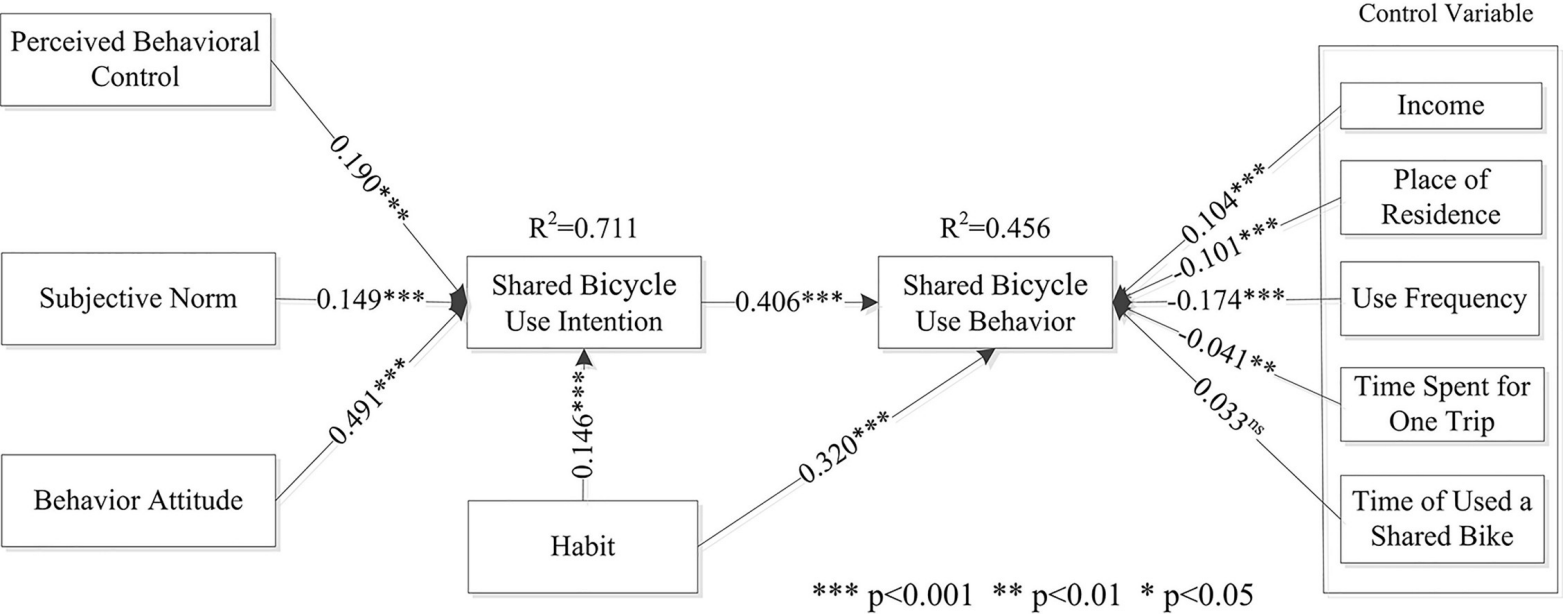
Results of structural model analysis. Pathways to shared bicycle use behavior through perceived behavioral control, subjective norm, behavior attitude, shared bicycle use intention, and habit.

Fig 2 shows that perceived behavioral control had a positive effect on shared bicycle use intention (β = 0.190, t = 11.110), confirming H1. Social norms also had a positive effect on shared bicycle use intention (β = 0.149, t = 6.771), confirming H2. Results showed that the behavior attitude of users had a positive effect on shared bicycle use intention (β = 0.491, t = 24.569), confirming H3. We found that shared bicycle use intention had a positive effect on shared bicycle use behavior (β = 0.406, t = 15.936), confirming H4. Finally, users’ habits had a positive effect on their shared bicycle use intention (β = 0.146, t = 7.226), confirming H5. The impact of users’ habits on their shared bicycle use behavior was also significant (β = 0.320, t = 11.921), confirming H6.

Control variables also showed significant associations with user’s intention and behavior. Monthly income positively affected shared bicycle use behavior (β = 0.104, t = 5.732). Residents living in urban areas were more likely to use shared bicycles (β = -0.101, t = 5.902). Frequency of shared bicycle use negatively affected self-reported shared bicycle use behavior(β = -0.174, t = 11.267). Time spent on a shared bicycle per trip negatively affected shared bicycle use behavior (β = -0.041, t = 2.469). Finally, the effect of time using a shared bicycle on shared bicycle use behavior was not significant (β = 0.033, t = 1.943).

### Moderation effects

Moderating roles can be tested by accessing differences in path coefficients for each subgroup [42, 43]. To examine differences in user characteristics, path comparison testing was conducted between groups. In this study, we dichotomized gender groups as male (group 1) and female (group 2), age groups as younger (age< = 30, group 1) and older (age>30, group 2) [42] and traveling distance groups as short distance (distance< = 2 km, group 1) and remote (distance>2 km, group 2)[44]. We then compared path coefficients based on the method of categorical moderating variables developed by Keil and Wassenaar [43].

#### Gender differences

The results indicated significant gender differences in shared bicycle users’ behavior intention and behavior (Table 6).

**Table 6:** Moderating effects of gender.

Items	Path	PC1	PC2	T	Remarks	Effect
Gender (N1 = Male) (N2 = Female)	PBC -> BI	0.266	0.128	43.965	O	(Male>Female)
	SN -> BI	0.095	0.177	-17.940	O	(Female>Male)
	AT -> BI	0.572	0.444	36.504	O	(Male>Female)
	HA -> BI	0.091	0.175	-20.920	O	(Female>Male)
	HA -> AB	0.359	0.247	33.054	O	(Male>Female)
	BI -> AB	0.381	0.436	-16.502	O	(Female>Male)

Note: $S_{pooled}=\sqrt{\left\{[(N_{1}-1)/(N_{1}+N_{2}-2)]\times SE_{1}^{2}+[(N_{2}-1)/(N_{1}+N_{2}-2)]\times SE_{2}^{2}\right\}}$.

$t=(PC_{1}-PC_{2})/[S_{Pooled}\times \sqrt{\left(1/N_{1}+1/N_{2})\right]}$, *N*_*i*_ = sample size of dataset for group *i*; SE_*i*_ = standard error of path in structural model of group *i*; PC_*i*_ = path coefficient in structural model of group *i*

Note: O: support; X: not support.

Specifically, the effect of perceived behavioral control and behavior attitude on shared bicycle use behavior intention was significantly higher for males than females. This implies that male users’ behavior intention increased more drastically with increase in perceived behavioral control and behavior attitude than female users. The interaction effects are plotted in S1 Fig and S2 Fig.

In contrast, the effect of subjective norms on shared bicycle use behavior intention was significantly higher in females. This implies that female users’ behavior intention increased more drastically with the increase in subjective norms (S3 Fig).

Furthermore, the effect of habits on shared bicycle use intention was significantly higher for females, while the effect of habits on shared bicycle use behavior was significantly higher in males. This implies that female users’ behavior intention increased more drastically with the increase in habits, and male users’ shared bicycle use behavior increased more drastically with the increase in habits (S4 Fig and S5 Fig).

Finally, the effect of shared bicycle use behavior intention on actual behavior was significantly higher in females. In other words, female users’ shared bicycle use behavior increased more drastically with the increase in behavior intention (S6 Fig).

#### Age differences

Age had a significant influence on shared bicycle use behavior intention and actual behavior (Table 7).

**Table 7:** Moderating effects of age.

Items	Path	PC1	PC2	T	Remarks	Effect
Age (N1 = Younger) (N2 = Older)	PBC -> BI	0.127	0.193	-17.860	O	(Older>Younger)
	SN -> BI	0.190	0.105	18.567	O	(Younger>Older)
	AT -> BI	0.527	0.504	5.854	O	(Younger>Older)
	HA -> BI	0.047	0.194	-33.307	O	(Older>Younger)
	HA -> AB	0.392	0.240	45.735	O	(Younger>Older)
	BI -> AB	0.156	0.573	-125.600	O	(Older>Younger)

Note: $S_{pooled}=\sqrt{\left\{[(N_{1}-1)/(N_{1}+N_{2}-2)]\times SE_{1}^{2}+[(N_{2}-1)/(N_{1}+N_{2}-2)]\times SE_{2}^{2}\right\}}$.

$t=(PC_{1}-PC_{2})/[S_{Pooled}\times \sqrt{\left(1/N_{1}+1/N_{2})\right]}$, *N*_*i*_ = sample size of dataset for group *i*; SE_*i*_ = standard error of path in structural model of group *i*; PC_*i*_ = path coefficient in structural model of group *i*

Note: O: support; X: not support.

The effect of perceived behavioral control and habit on shared bicycle use behavior intention was significantly higher for older users. This means that older users’ behavior intention increased more drastically with the increase in perceived behavioral control and habit than younger users (S7 Fig and S8 Fig).

On the other hand, the effect of subjective norms and behavior attitude on shared bicycle use behavior intention was significantly higher for younger users. This means that younger users’ behavior intention increased more drastically with the increase in subjective norms and behavior attitude than older users (S9 Fig and S10 Fig).

The effect of habits on shared bicycle travel actual behavior was significantly higher for younger users. In other words, younger users’ shared bicycle use behavior increased more drastically with the increase in habits. Finally, the effect of shared bicycle use intention on actual behavior with regard to shared bicycle travel was significantly higher for older users. This means that older users’ shared bicycle use behavior increased more drastically with the increase in behavior intention (S11 Fig and S12 Fig).

#### Distance differences

Distance traveled also influenced the formation of users’ intentions and behavior (Table 8).

**Table 8:** Moderating effects of distance.

Items	Path	PC1	PC2	T	Remarks	Effect
Distance (N1 = Short distance) (N2 = Remote)	PBC -> BI	0.271	0.151	35.157	O	(Short distance>Remote)
	SN -> BI	0.095	0.180	-18.072	O	(Remote>Short distance)
	AT -> BI	0.434	0.532	-25.285	O	(Remote>Short distance)
	HA -> BI	0.179	0.115	15.024	O	(Short distance>Remote)
	HA -> AB	0.279	0.386	-31.213	O	(Remote>Short distance)
	BI -> AB	0.384	0.423	-10.988	O	(Remote>Short distance)

Note: $S_{pooled}=\sqrt{\left\{[(N_{1}-1)/(N_{1}+N_{2}-2)]\times SE_{1}^{2}+[(N_{2}-1)/(N_{1}+N_{2}-2)]\times SE_{2}^{2}\right\}}$.

$t=(PC_{1}-PC_{2})/[S_{Pooled}\times \sqrt{\left(1/N_{1}+1/N_{2})\right]}$, *N*_*i*_ = sample size of dataset for group *i*; SE_*i*_ = standard error of path in structural model of group *i*; PC_*i*_ = path coefficient in structural model of group *i*

Note: O: support; X: not support.

The effect of perceived behavioral control and habits on shared bicycle use behavior intention was significantly higher for short distance users. This means that short distance users’ behavior intention increased more drastically with the increase in perceived behavioral control and habit (S13 Fig and S14 Fig).

In contrast, the effect of subjective norms and attitude on shared bicycle use behavior intention was significantly higher for remote users. This tells us that remote users’ behavior intention increased more drastically with the increase in subjective norms and behavior attitude (S15 Fig and S16 Fig).

Finally, the effect of habits and behavior intention on shared bicycle travel actual behavior was significantly higher for remote users. In other words, remote users’ shared bicycle use behavior increased more drastically with the increase in habits and behavior intention (S17 Fig and S18 Fig).

## Discussion

### Key findings

Based on a case study of shared bicycle use in China, we investigated the factors potentially influencing shared bicycle use behavior. Through the analysis of data obtained from an online questionnaire, we explored a range of demographic variables and situational factors affecting shared bicycle travel use. In the following, we summarize and discuss our main findings.

First, the behavioral intention to adopt shared bicycle use for bicycle travel was significantly influenced by four variables: attitude, social norms, perceived behavioral control and travel habits. Relying on the framework of the theory of planned behavior, Chen [45] found that the perceived pleasure in using public bikes had a strong influence on sustainable continuous use of public bikes for users in Taipei, and that subjective norms were more effective for non-users. Moreover, for residents in Denmark using bike-sharing frequently and for multiple purposes during holidays, Kaplan et al. [46] pointed out that their attitudes, subjective norms toward cycling, past cycling experience, habitual transport mode choice, and holiday partners influenced their frequent and multi-purpose cycling intentions. Our report extends the framework of the theory of planned behavior by confirming that attitude, social norms, perceived behavioral control and travel habits are important factors affecting users’ intentions to adopt shared bicycle use for bicycle travel in daily life in China. Most importantly, we found that amongst the variables, users’ attitudes towards shared bicycle travel had the greatest impact. This conclusion helps us to form a more complete understanding of the path formation of shared bike users’ behavioral intention. These findings would imply that shared bicycle companies should focus their efforts on environmental publicity, on promoting the concept of shared bicycle travel amongst users, and on convincing potential users that shared bicycle use serves both individual and collective interests. Those initiatives should contribute to the emergence of a more positive attitude towards shared bicycle travel.

Second, shared bicycle use behavior was significantly influenced by both shared bicycle use intention and habits, although the impact of shared bicycle use intention was stronger. Donald et al. [33] found that attitudes, perceived behavioral control and subjective norms increased an individual’s habit and intentions to drive to work, while environmental concerns reduced a person’s tendency to drive. In turn, both habits and intentions increase the likelihood that a person will drive rather than travel by bicycle. This paper confirmed that shared bicycle use intention and habits also positively affect shared bicycle use behavior. In addition, we contribute to the theory of planned behavior by verifing that the impact of shared bicycle use intention was more influential. Prior research showed that behavioral intentions played a leading role in behavioral choices in a new environment[24]. This implies that shared bicycles travel is a relatively new feature in Chinese urban environments, and therefore needs to be consolidated as a new behavioral habit. Marketing measures, such as coupons, can be used to reinforce bike sharing users’ habit formation. At the same time, analyses of control variables showed that monthly income positively affected the tendency to travel by means of a shared bicycle. This conclusion was also proved by Murphy and Usher [47], who found that bicycle sharing was predominantly used by higher-income individuals in Dublin, Ireland. One of the possible explanations may be that higher-income users tend to favor bicycling for enjoyment and exercise. Results showed that residence location had a significant negative impact on shared bicycle use, perhaps because the farther away from the city people live, the fewer the number of bikes shared and the lower their usage. Travel time negatively affected shared bicycle use because when the distance was far, people often used public transportation or their own vehicles [48]. Finally, it is interesting to note that the frequency of bicycle use negatively affected shared bicycle use behavior. One of the reasons may be that younger users want to try new things and several new alternative options have appeared such as shared cars powered by storage-batteries. Some users of shared bicycles have had bad experiences [49] and this can have strong negative effects on overall use.

Third, this paper contributes to the TPB model by identifing significant effects of demographic variables (gender and age) and situational factors (distance). The influence of gender on mode of travel has been examined in previous studies[50, 51] and research showed that women were more concerned about the cost of using a car and parking and, therefore were more likely to use public transportation and leave the car at home [52]. Zhao et al. [53] reported that women were more likely than men to make multiple (two or more) bike-sharing trips from one origin to a single destination and then back to the same origin, especially on weekdays. Similar to the findings from other public transportation behaviour domains[54, 55], this paper contributes to the theory of planned behavior by verifing that male users were most likely to choose shared bicycle travel when they perceived behavioral control and attitudes, while female users were most likely to choose shared bicycle travel because of perceived subjective norms and travel habits. The research conclusions of this paper are basically consistent with those of previous studies in different situations [54, 55]. Age also plays an important role in users’ travel behavior [56]. Prior investigations showed that age was significantly correlated with substitutive walking behavior [57], and Barnes et al. [58] averred that transit access may be especially important in older age groups and walkability may be especially important for middle-aged and older adults who are still working. Similar to the findings from other public transportation behaviour domains[55, 59], we found that older bicycle users were most likely to travel by shared bicycle because of perceived behavioral control and travel habits, while younger users were most likely to use shared bicycles due to subjective norms and attitudes. To the best of our knowledge, this is the first paper to examine age differences when considering shared bicycle travel within the framework of the theory of planned behavior. Our research conclusion is a useful supplement to the theory of planned behavior. Finally, the trip distance has been shown to affect travel mode choice [60, 61]. Xing et al. [62] pointed out that transportation cycling was most often used for short distances to utilitarian destinations such as the grocery store or post office, while long distances and safe routes more often involved recreational cycling. In this report we noted that short-distance travelers were most likely to choose shared bicycle travel for reasons of perceived behavioral control and travel habits, while for long-distance users, subjective norms and attitudes were most likely to influence their choice to use shared bicycle travel. Our research results provide a micro-knowledge base for the factors that influence users’ choice to use shared bicycle travel, and also deepens the understanding of shared bicycle travel. Taken together, these findings suggest that marketing initiatives for shared bicycles should focus on the factors specifically influencing demographic groups differentiated by gender, age, and travel distance and introduce personalized shared bicycle travel options to meet the needs of specific groups.

Fourth, our online survey also recorded suggestions from users to current bicycle sharing operators. Those included increasing the number of shared bicycles, increasing bicycle parking spots, enactment of new regulations to strengthen management of shared bicycle parking, and improving bicycle inspection and repair. Some users proposed that the bicycle models were not diversified enough, and thus not suitable for all people. Modifications aiming to make the service more user-friendly, such as increasing the height of child seats, and increasing the size of water cup holders were also suggested and should be considered. The survey indicated that shared bicycle operators should pay attention to problems raised by users and act as soon as possible.

### Implications

Operators of shared bicycle services should consider the perceptions, perceived behavioral control, subjective norms, travel attitudes and habits of users when defining the next steps in their efforts to popularize shared bicycle travel. Initiatives should include additional shared bicycle travel marketing campaigns, and increased initiatives to change users’ travel behavior by influencing their beliefs, attitudes and values[63]. At the same time, service operators should take into account the different demographic characteristics of users. Operators should also consolidate delivery and maintenance of shared bicycles, and create better regulations for bicycle parking and placement. Moreover, users could contribute to a solution to the problems of traffic congestion and smog by adopting shared bicycle use and embracing shared bicycle travel. From the perspective of governments, shared bicycle use provides a simple, economic and efficient solution to heavy traffic and environmental pressures in urban areas [64]. Therefore, authorities should implement initiatives to promote shared bicycle use, increase the effort to guide public opinion towards shared bicycle use, and build a shared bicycle travel atmosphere with the help of social networking and public media.

### Limitations

Like other empirical studies, there are limitations to this research which should be considered when discussing the results. First, an online survey method was used in our study, which reflects respondents’ subjective perceptions towards the investigated questions. Subjective data has some inherent drawbacks that are hard to avoid in surveys [65]. In this regard, objective results such as archival data may help to provide additional insights for specific research. Our data were gathered during a single time period. Cross-sectional data do not allow for a dynamic assessment of changes in the intentions and related behavior of users, which may affect the applicability of our results. Future research should investigate the intentions and behavior of shared bicycle travel users through a combination of cross-sectional and longitudinal research. Secondly, based on the theory of planned behavior, this study focused on the effects of travel attitude, social norms, perceived behavioral control and travel habits on mechanisms of shared bicycle use intention and behavior. Some other factors were omitted, such as the potentially moderating impact that a person's environmental world view or personal norms [66] could have upon their intentions and behaviour within our model. Our outcome measures, including shared bicycle use intention and behavior, were evaluated by self-reported data collection and, therefore, it is possible that some of the variance in self-reported shared bicycle use behavior could reflect perceived behavior or response biases rather than actual behavior [67, 68]. Thirdly, future studies should also evaluate the effects of additional factors not included in our analysis, such as the level of comfort provided by current bicycle design, bicycle layout optimization, or bicycle model options such as electric motor assists. Finally, our survey was conducted in a single cultural context (China) and a more general statement could be made by applying the developed research model and research conclusions to other countries and cultures [69].

## Conclusions

Shared bicycle services have significantly expanded in China. However, few studies have specifically addressed the factors underlying the decision-making process behind shared bicycle use. To fill this research gap, this study used the theory of planned behaviour as a framework for understanding the public views on shared bicycles as transportation options. The results indicated that shared bicycle users’ travel attitudes, social norms, travel habits and perceived behavioral control significantly affect their behavioral intentions and actual behavior. More importantly, our results indicated that shared bicycle users’ behavioral intentions and actual behavior showed significant differences between different demographic variables (gender, age) and situational factors (distance). These findings have advanced the theory of planned behaviour and enriched the literature on shared bicycle services. The conclusions of this study provide useful information for bicycle service operators and government policy makers. It should be reiterated that the sample size in this study was small; therefore, it would be beneficial to repeat this study using a larger sample group from a more diverse population.

## Supporting information

S1 Fig Moderating effect of gender between perceived behavioral control and behavior intention. (TIF)

S2 Fig Moderating effect of gender between behavior attitude and behavior intention. (TIF)

S3 Fig Moderating effect of gender between subjective norms and behavior intention. (TIF)

S4 Fig Moderating effect of gender between habits and behavior intention. (TIF)

S5 Fig Moderating effect of gender between habits and shared bicycle use behavior. (TIF)

S6 Fig Moderating effect of gender between behavior intention and shared bicycle use behavior. (TIF)

S7 Fig Moderating effect of age between perceived behavioral control and behavior intention. (TIF)

S8 Fig Moderating effect of age between behavior attitude and behavior intention. (TIF)

S9 Fig Moderating effect of age between subjective norms and behavior intention. (TIF)

S10 Fig Moderating effect of age between habits and behavior intention. (TIF)

S11 Fig Moderating effect of age between habits and shared bicycle use behavior. (TIF)

S12 Fig Moderating effect of age between behavior intention and shared bicycle use behavior. (TIF)

S13 Fig Moderating effect of distance between perceived behavioral control and behavior intention. (TIF)

S14 Fig Moderating effect of distance between behavior attitude and behavior intention. (TIF)

S15 Fig Moderating effect of distance between subjective norms and behavior intention. (TIF)

S16 Fig Moderating effect of distance between habits and behavior intention. (TIF)

S17 Fig Moderating effect of distance between habits and shared bicycle use behavior. (TIF)

S18 Fig Moderating effect of distance between behavior intention and shared bicycle use behavior. (TIF)

S1 File Data. (XLS)

## References

[1] Tao M, Chen L, Xiong X, Zhang M, Ma P, Tao J, et al Formation process of the widespread extreme haze pollution over northern China in January 2013: Implications for regional air quality and climate. Atmospheric Environment. 2014;98:417–25.

[2] Hart PS. One or many? the influence of episodic and thematic climate change frames on policy preferences and individual behavior change. Science Communication. 2011;32(1):28–51.

[3] Zhang X, Ma L, Wang GS. Factors influencing users’ subjective well-being: an empirical study based on shared bicycles in China. Information Discovery and Delivery. 2017;45(4):202–11.

[4] Ma L, Zhang X, Wang GS. Identifying the reasons why users in China recommend bike apps. International Journal of Market Research. 2017;59(6):767–86.

[5] CNNIC. Forty-two statistical report on the development of China Internet network 2018. Available from: http://www.cnnic.net.cn/.

[6] Zhang L, Zhang J, Duan ZY, Bryde D. Sustainable bike-sharing systems: characteristics and commonalities across cases in urban China. Journal of Cleaner Production. 2015;97:124–33.

[7] Hung K, Petrick JF. Testing the effects of congruity, travel constraints, and self-efficacy on travel intentions: An alternative decision-making model. Tourism Management. 2012;33(4):855–67.

[8] Chaney RA, Sloan CD, Cooper VC. Personal exposure to fine particulate air pollution while commuting: An examination of six transport modes on an urban arterial roadway. Plos One. 2017;12(11):e0188053 10.1371/journal.pone.0188053 . 29121096 PMC5679559

[9] Huang Y, Hsu MK, Swanson S. Grocery store image, travel distance, satisfaction and behavioral intentions. International Journal of Retail & Distribution Management. 2010;38(2):115–32.

[10] Klöckner CA, Friedrichsmeier T. A multi-level approach to travel mode choice–How person characteristics and situation specific aspects determine car use in a student sample. Transportation Research Part F Traffic Psychology & Behaviour. 2011;14(4):261–77.

[11] Hsiao CH, Yang C. Predicting the travel intention to take high speed rail among college students. Transportation Research Part F Psychology & Behaviour. 2010;13(4):277–87.

[12] Li MM, Cai LA. The effects of personal values on travel motivation and behavioral intention. Journal of Travel Research. 2012;51(4):473–87.

[13] Kuo NW, Dai YY. Applying the theory of planned behavior to predict low-carbon tourism behavior: A modified model from Taiwan: IGI Global; 2012 45–62.

[14] Schwanen T, Banister D, Anable J. Rethinking habits and their role in behaviour change: the case of low-carbon mobility. Journal of Transport Geography. 2012;24(4):522–32.

[15] Line T, Chatterjee K, Lyons G. The travel behaviour intentions of young people in the context of climate change. Journal of Transport Geography. 2010;18(2):238–46.

[16] Guo Y, Zhou J, Wu Y, Li Z. Identifying the factors affecting bike-sharing usage and degree of satisfaction in Ningbo, China. Plos One. 2017;12(9):e0185100 10.1371/journal.pone.0185100 . 28934321 PMC5608320

[17] Yasuoka J, Nanishi K, Kikuchi K, Suzuki S, Ly P, Thavrin B, et al Barriers for pregnant women living in rural, agricultural villages to accessing antenatal care in Cambodia: A community-based cross-sectional study combined with a geographic information system. Plos One. 2018;13(3):e0194103 10.1371/journal.pone.0194103 . 29554118 PMC5858830

[18] Fishbein M, Ajzen I. Belief, attitude, intention and behaviour: an introduction to theory and research. Philosophy & Rhetoric. 1975;41(4):842–4.

[19] Mathieson K. Predicting user intentions: comparing the technology acceptance model with the theory of planned behavior. Information Systems Research. 1991;2(3):173–91.

[20] Azjen I. The theory of planned behavior. British Journal of Social Psychology. 1991;40(4):471–499. 10.1348/014466601164939 11795063

[21] Ajzen I. The theory of planned behaviour: reactions and reflections. Psychology & Health. 2011;26(9):1113–1127. 21929476 10.1080/08870446.2011.613995

[22] Jun J, Arendt SW. Understanding healthy eating behaviors at casual dining restaurants using the extended theory of planned behavior. International Journal of Hospitality Management. 2016;53:106–15.

[23] Han HS, Hsu LT, Sheu C. Application of the theory of planned behavior to green hotel choice: Testing the effect of environmental friendly activities. Tourism Management. 2010;31(3):325–34.

[24] Liu D, Du H, Southworth F, Ma S. The influence of social-psychological factors on the intention to choose low-carbon travel modes in Tianjin, China. Transportation Research Part A Policy & Practice. 2017;105:42–53.

[25] Klockner CA, Blobaum A. A comprehensive action determination model: Toward a broader understanding of ecological behaviour using the example of travel mode choice. Journal of Environmental Psychology. 2010;30(4):574–86.

[26] Neal DT, Wood W, Labrecque JS, Lally P. How do habits guide behavior? Perceived and actual triggers of habits in daily life. Journal of Experimental Social Psychology. 2012;48(2):492–8.

[27] Polites GL, Karahanna E. The embeddedness of information systems habits in organizational and individual level routines: Development and disruption. Mis Quarterly. 2013;37(1):221–46.

[28] Fu X, Juan Z. Understanding public transit use behavior: integration of the theory of planned behavior and the customer satisfaction theory. Transportation. 2017; 44 (5):1–22.

[29] Huma ZE, Hussain S, Thurasamy R, Malik MI. Determinants of cyberloafing: a comparative study of a public and private sector organization. Internet Research. 2017;27(1):97–117.

[30] Chen CF, Chao WH. Habitual or reasoned? Using the theory of planned behavior, technology acceptance model, and habit to examine switching intentions toward public transit. Transportation Research Part F Traffic Psychology & Behaviour. 2011;14(2):128–37.

[31] Towler G, Shepherd R. Modification of Fishbein and Ajzen's theory of reasoned action to predict chip consumption. Food Quality & Preference. 1991;3(1):37–45.

[32] Jäppinen S, Toivonen T, Salonen M. Modelling the potential effect of shared bicycles on public transport travel times in Greater Helsinki: An open data approach. Applied Geography. 2013;43(43):13–24.

[33] Donald IJ, Cooper SR, Conchie SM. An extended theory of planned behaviour model of the psychological factors affecting commuters' transport mode use. Journal of Environmental Psychology. 2014;40:39–48.

[34] Chen IYL. The factors influencing members' continuance intentions in professional virtual communities-a longitudinal study. Journal of Information Science. 2010;33(4):451–67.

[35] Khalifa M, Liu V. Online consumer retention: contingent effects of online shopping habit and online shopping experience. European Journal of Information Systems. 2007;16(6):780–92.

[36] Courtois C, Montrieux H, Grove FD, Raes A, Marez LD, Schellens T. Student acceptance of tablet devices in secondary education: A three-wave longitudinal cross-lagged case study. Computers in Human Behavior. 2014;35(35):278–86.

[37] Ma L, Zhang X, Ding X, Wang G. Bike sharing and users' subjective well-being: An empirical study in China. Transportation Research Part A Policy & Practice. 2018;118:14–24.

[38] Zhang X, Ma L, Wang G-S. Investigating consumer word-of-mouth behaviour in a Chinese context. Total Quality Management & Business Excellence. 2017:1–15. 10.1080/14783363.2017.1317587

[39] Chen X, Ma J, Jin J, Fosh P. Information privacy, gender differences, and intrinsic motivation in the workplace. International Journal of Information Management. 2013;33(6):917–26.

[40] Soper DS. A-priori sample size calculator for structural equation models [Software] 2013 Available from: http://www.danielsoper.com/statcalc.

[41] Hair JF, Black WC, Babin BJ, Anderson RE. Multivariate data analysis(7th Edition). New Jersey: Prentice Hall; 2009.

[42] Ma Liang, Zhang Xin, Ding XY. Social media users’ share intention and subjective well-being: An empirical study based on WeChat. Online Information Review. 2018;(6):1–18.

[43] Keil M, Wassenaar A. A cross-cultural study on escalation of commitment behavior in software projects. Mis Quarterly. 2000;24(2):299–325.

[44] Nicolau JL, Más FJ. The influence of distance and prices on the choice of tourist destinations: the moderating role of motivations. Tourism Management. 2006;27(5):982–96.

[45] Chen SY. Using the sustainable modified TAM and TPB to analyze the effects of perceived green value on loyalty to a public bike system. Transportation Research Part A Policy & Practice. 2016;88:58–72.

[46] Kaplan S, Manca F, Nielsen TAS, Prato CG. Intentions to use bike-sharing for holiday cycling: An application of the Theory of Planned Behavior. Tourism Management. 2015;47:34–46.

[47] Murphy E, Usher J. The role of bicycle-sharing in the city: Analysis of the Irish experience. International Journal of Sustainable Transportation. 2015;9(2):116–25.

[48] Ji Y, Fan Y, Ermagun A, Cao X, Wang W, Das K. Public bicycle as a feeder mode to rail transit in China: The role of gender, age, income, trip purpose, and bicycle theft experience. International Journal of Sustainable Transportation. 2017;11(4):308–17.

[49] Hackbarth G, Grover V, Yi MY. Computer playfulness and anxiety: positive and negative mediators of the system experience effect on perceived ease of use. Information & Management. 2003;40(3):221–32.

[50] Gordon P, Kumar A, Richardson HW. Gender differences in Metropolitan travel behaviour. Regional Studies. 1989;23(6):499–510.

[51] Elias W, Benjamin J, Shiftan Y. Gender differences in activity and travel behavior in the Arab world. Transport Policy. 2015;44(1):19–27.

[52] Simićević J, Milosavljević N, Djoric V. Gender differences in travel behaviour and willingness to adopt sustainable behaviour. Transportation Planning & Technology. 2016;39(5):1–11.

[53] Zhao J, Wang J, Deng W. Exploring bikesharing travel time and trip chain by gender and day of the week. Transportation Research Part C. 2015;58:251–64.

[54] Moan IS. Whether or not to ride with an intoxicated driver: Predicting intentions using an extended version of the theory of planned behaviour. Transportation Research Part F Traffic Psychology & Behaviour. 2013;20(3):193–205.

[55] Morris MG, Venkatesh V, Ackerman PL. Gender and age differences in employee decisions about new technology: an extension to the theory of planned behavior. IEEE Transactions on Engineering Management. 2005;52(1):69–84.

[56] Irawan MZ, Sumi T. Promoting active transport in students’ travel behavior: A case from Yogyakarta (Indonesia). Journal of Sustainable Development. 2011;4(1):45–52.

[57] Piatkowski DP, Krizek KJ, Handy SL. Accounting for the short term substitution effects of walking and cycling in sustainable transportation. Travel Behaviour & Society. 2015;2(1):32–41.

[58] Barnes R, Winters M, Ste-Marie N, Mckay H, Ashe MC. Age and retirement status differences in associations between the built environment and active travel behaviour. Journal of Transport & Health. 2016;3(4):513–22.

[59] Baker EW, Al-Gahtani SS, Hubona GS. The effects of gender and age on new technology implementation in a developing country. Information Technology & People. 2007;20(4):352–75.

[60] Hunecke M, Haustein S, Grischkat S, Böhler S. Psychological, sociodemographic, and infrastructural factors as determinants of ecological impact caused by mobility behavior. Journal of Environmental Psychology. 2007;27(4):277–92.

[61] Wolf A, Seebauer S. Technology adoption of electric bicycles: A survey among early adopters. Transportation Research Part A. 2014;69:196–211.

[62] Xing Y, Handy SL, Mokhtarian PL. Factors associated with proportions and miles of bicycling for transportation and recreation in six small US cities. Transportation Research Part D Transport & Environment. 2010;15(2):73–81.

[63] Li Z. Behavioural implications of preferences, risk attitudes and beliefs in modelling risky travel choice with travel time variability. Transportation. 2013;40(3):505–23.

[64] El-Assi W, Mahmoud MS, Habib KN. Effects of built environment and weather on bike sharing demand: a station level analysis of commercial bike sharing in Toronto. Transportation. 2017;44(3):589–613.

[65] Pakpour AH, Gellert P, Asefzadeh S, Updegraff JA, Molloy GJ, Sniehotta FF. Intention and planning predicting medication adherence following coronary artery bypass graft surgery. Journal of Psychosomatic Research. 2014;77(4):287–95. 10.1016/j.jpsychores.2014.07.001 25280826

[66] Harland P, Staats H, Wilke HAM. Explaining proenvironmental intention and behavior by personal norms and the theory of planned behavior. Journal of Applied Social Psychology. 1999;29(12):2505–28.

[67] Lin CY, Updegraff JA, Pakpour AH. The relationship between the theory of planned behavior and medication adherence in patients with epilepsy. Epilepsy & Behavior. 2016;61:231–6. 27390026 10.1016/j.yebeh.2016.05.030

[68] Strong C, Lin CY, Jalilolghadr S, Updegraff JA, Broström A, Pakpour AH. Sleep hygiene behaviours in Iranian adolescents: an application of the Theory of Planned Behavior. Journal of Sleep Research. 2018;27(1):23–31. 10.1111/jsr.12566 28593637

[69] Sun Y, Fang Y, Lim KH, Straub D. User satisfaction with information technology services: A social capital perspective. Information Systems Research. 2012;23(4):1195–211.

