# Supplementary figures and images for: Psychosocial factors influencing shared bicycle travel choices among Chinese: An application of theory planned behavior
Source: PLoS One. 2019 Jan 25;14(1):e0210964. doi: 10.1371/journal.pone.0210964 (PMC6347275; doi:10.1371/journal.pone.0210964)

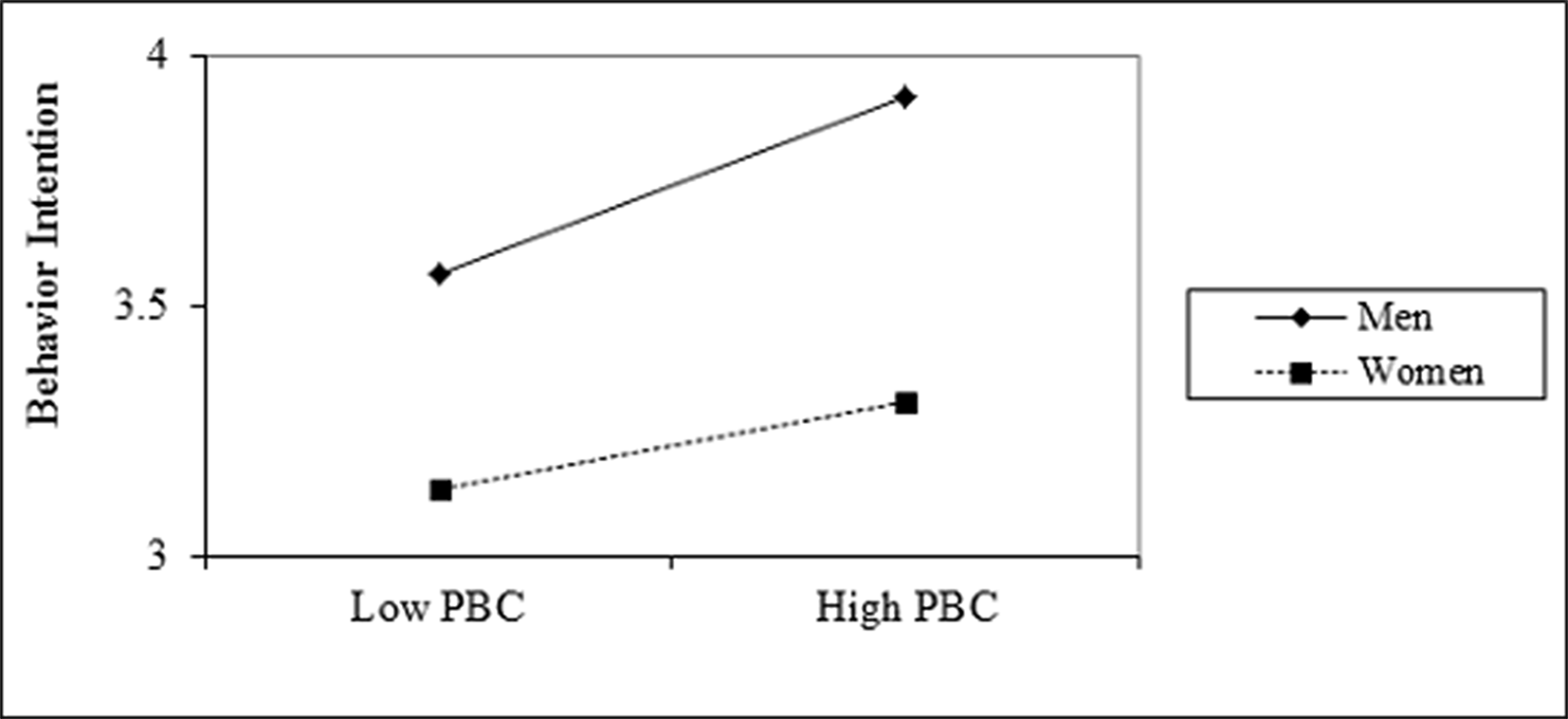

Supplement: S1 Fig — (TIF) [file pone.0210964.s001.tif]

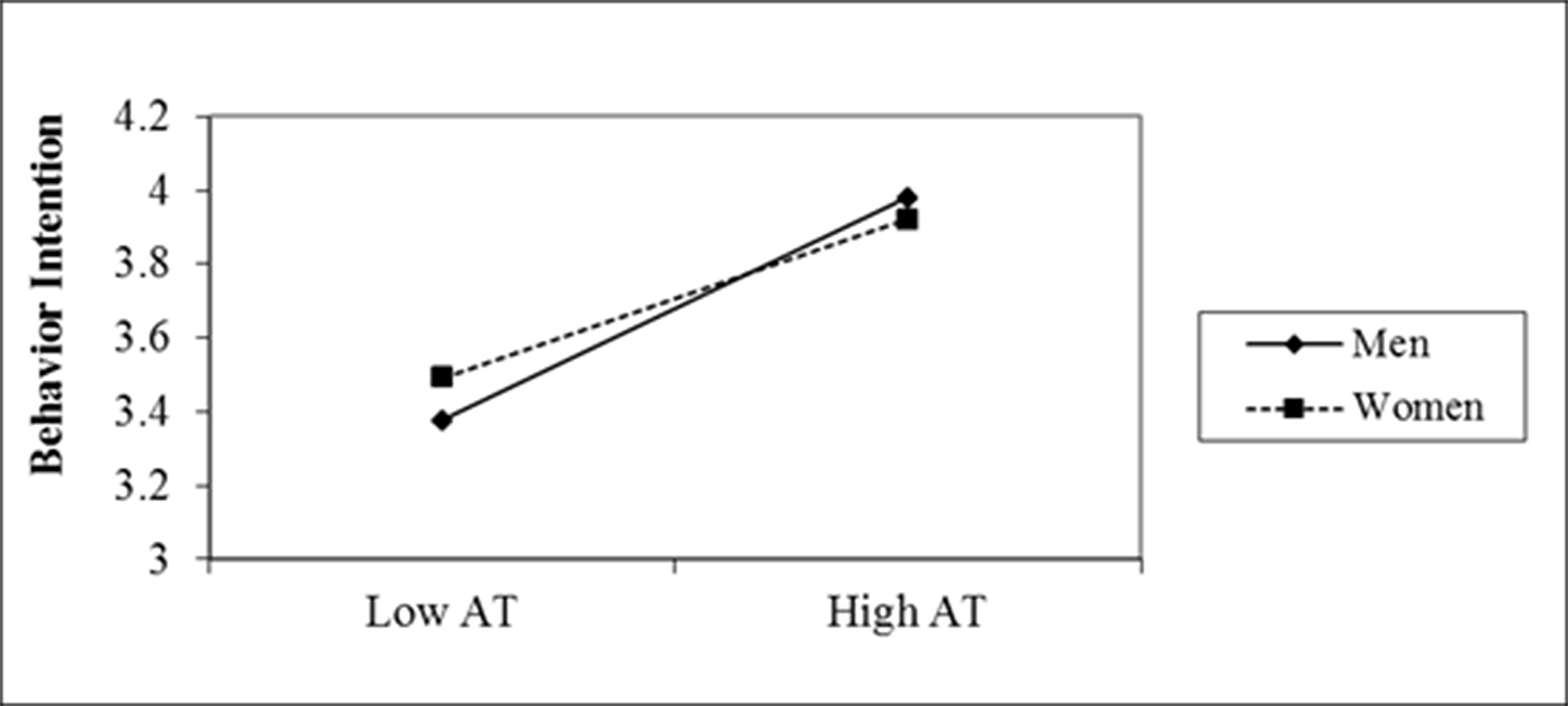

Supplement: S2 Fig — (TIF) [file pone.0210964.s002.tif]

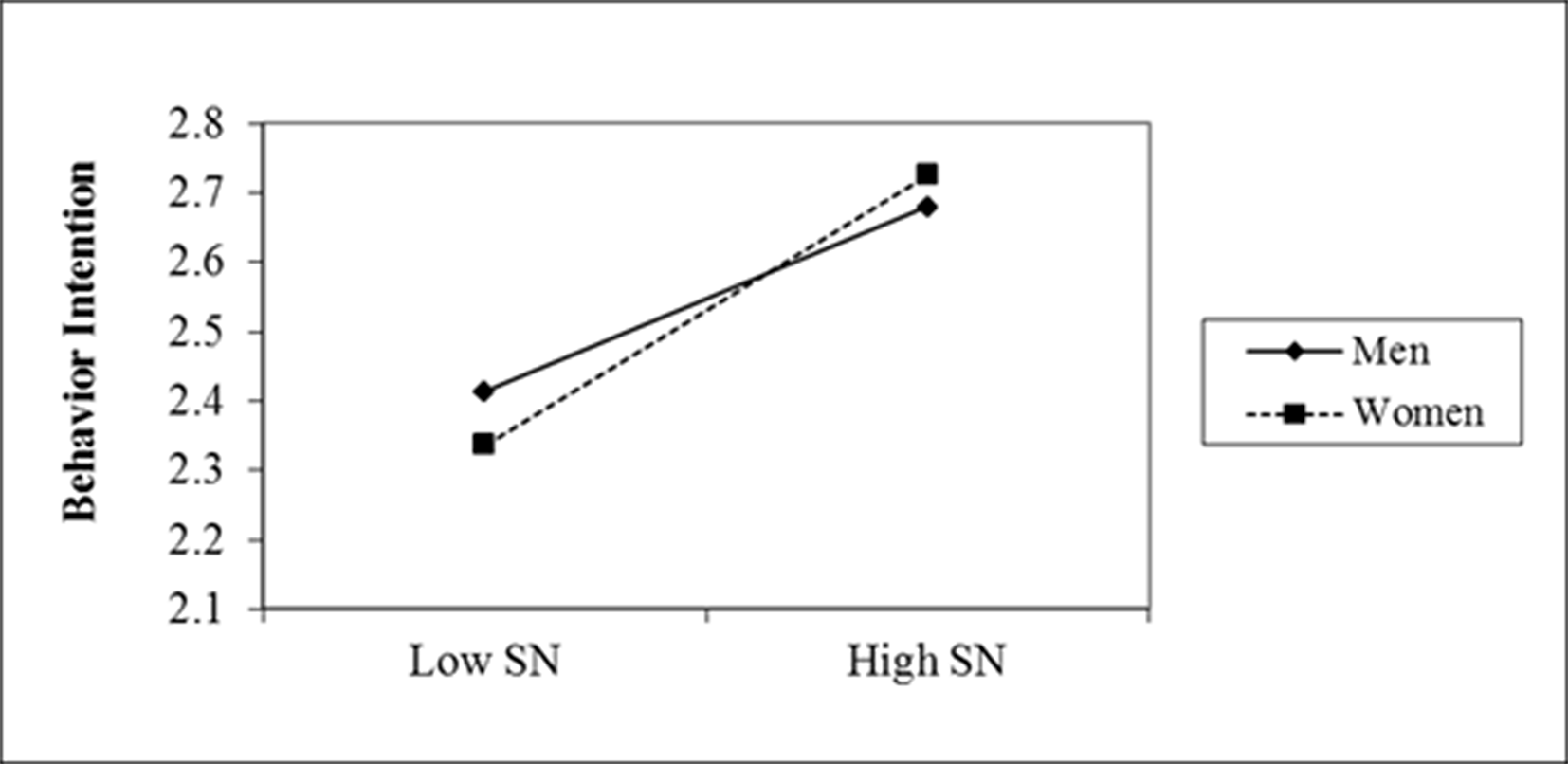

Supplement: S3 Fig — (TIF) [file pone.0210964.s003.tif]

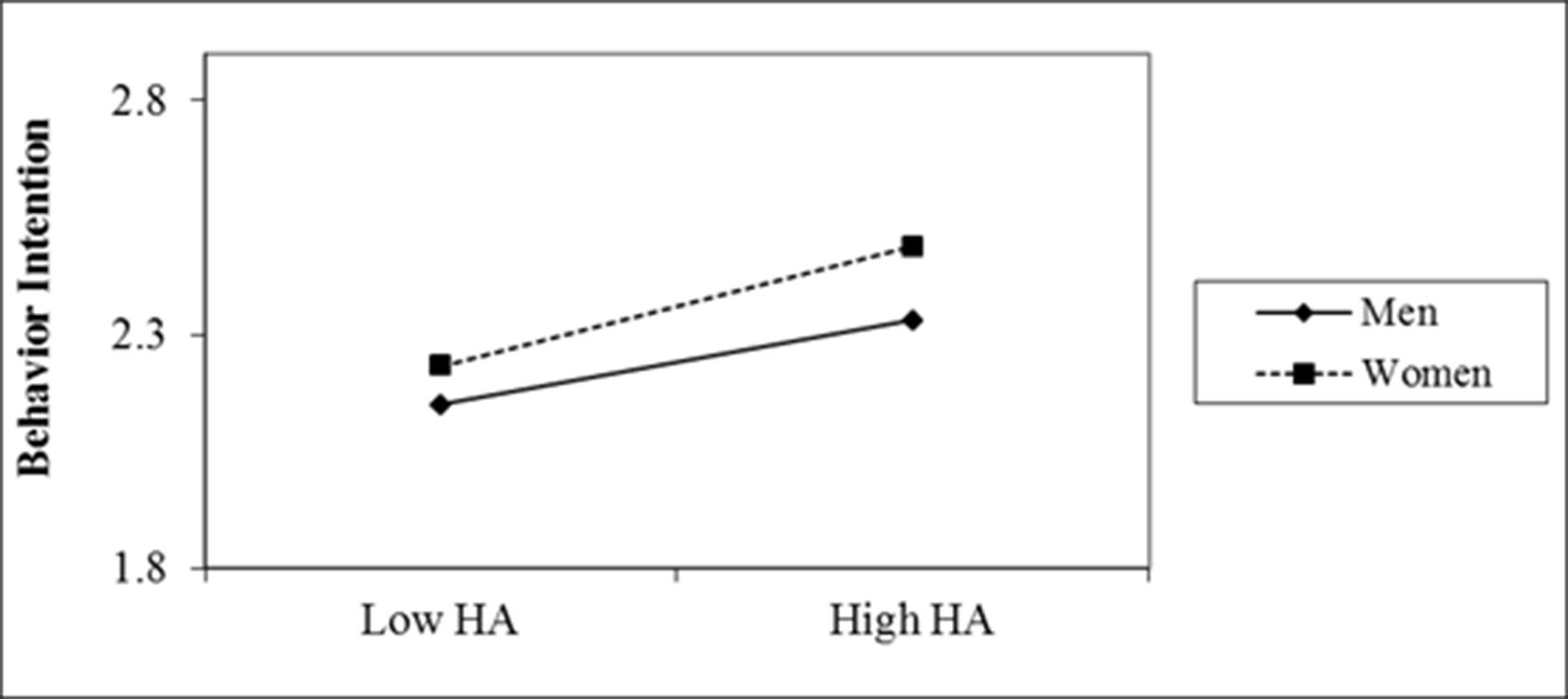

Supplement: S4 Fig — (TIF) [file pone.0210964.s004.tif]

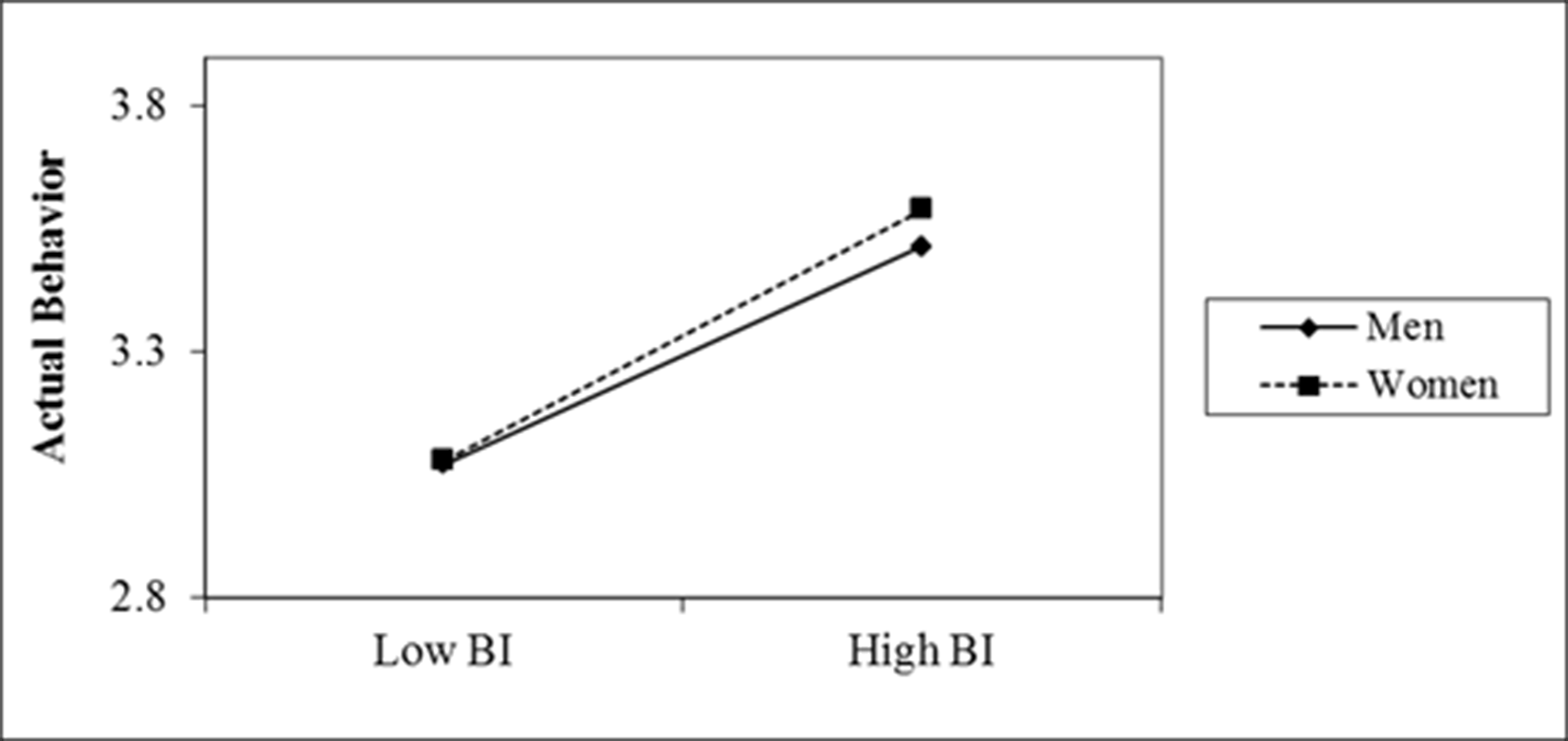

Supplement: S5 Fig — (TIF) [file pone.0210964.s005.tif]

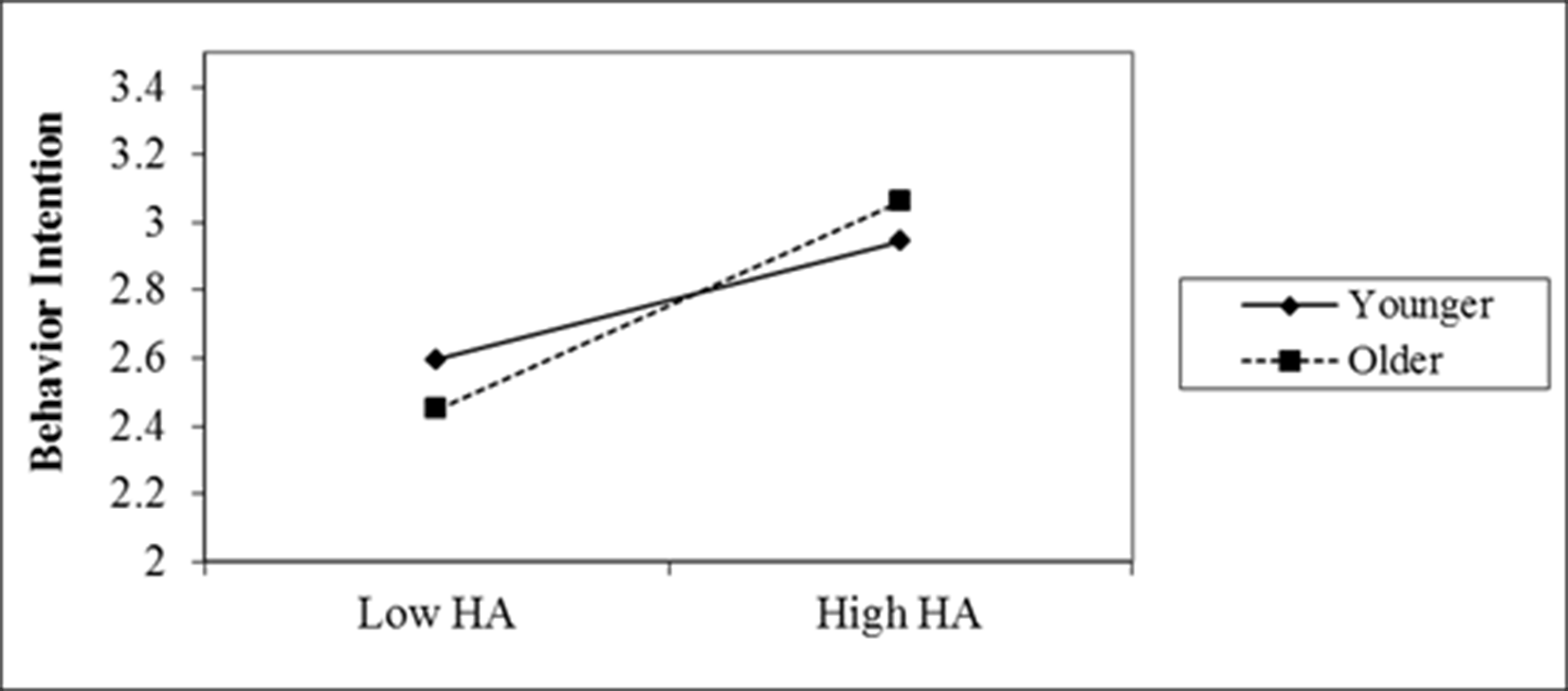

Supplement: S7 Fig — (TIF) [file pone.0210964.s007.tif]

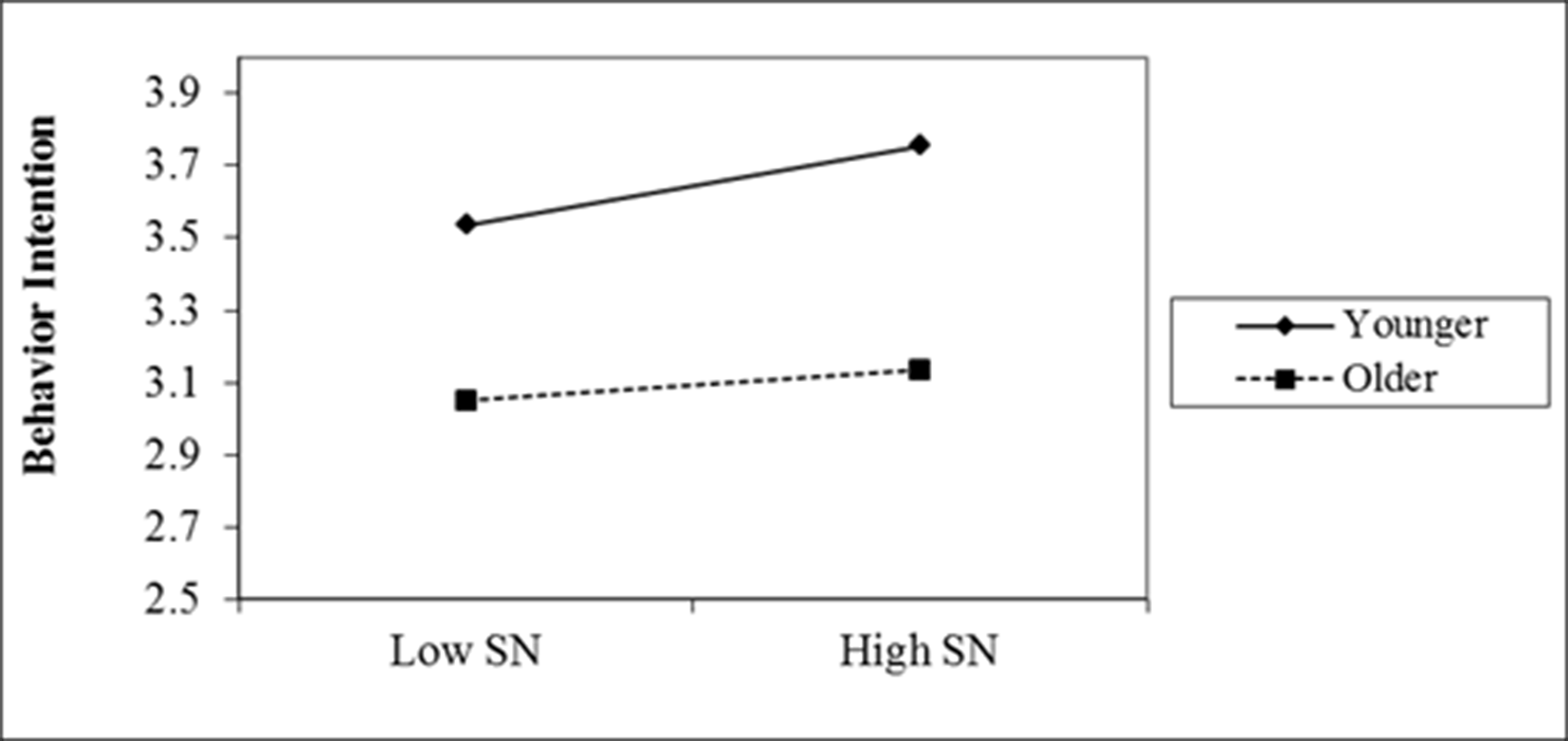

Supplement: S8 Fig — (TIF) [file pone.0210964.s008.tif]

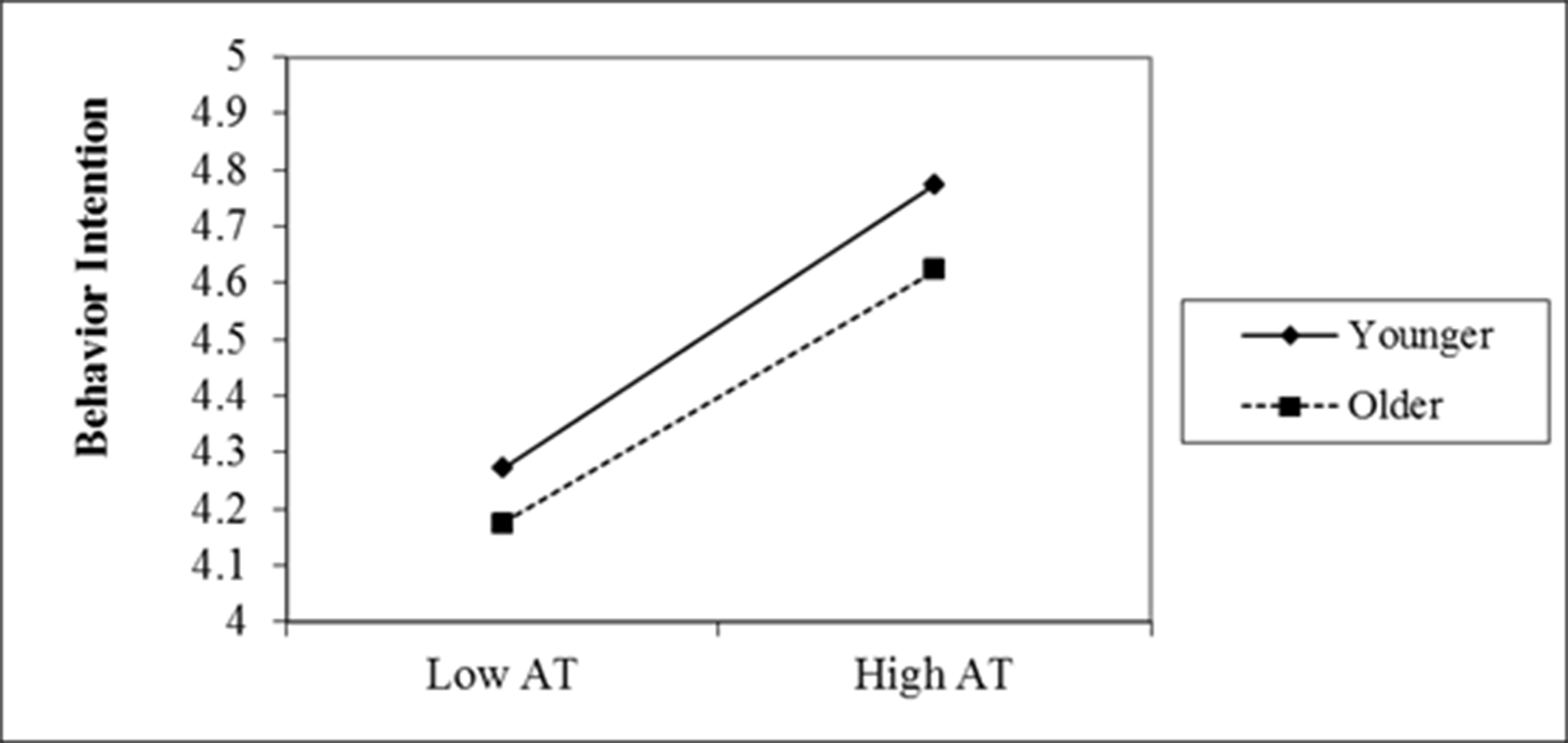

Supplement: S9 Fig — (TIF) [file pone.0210964.s009.tif]

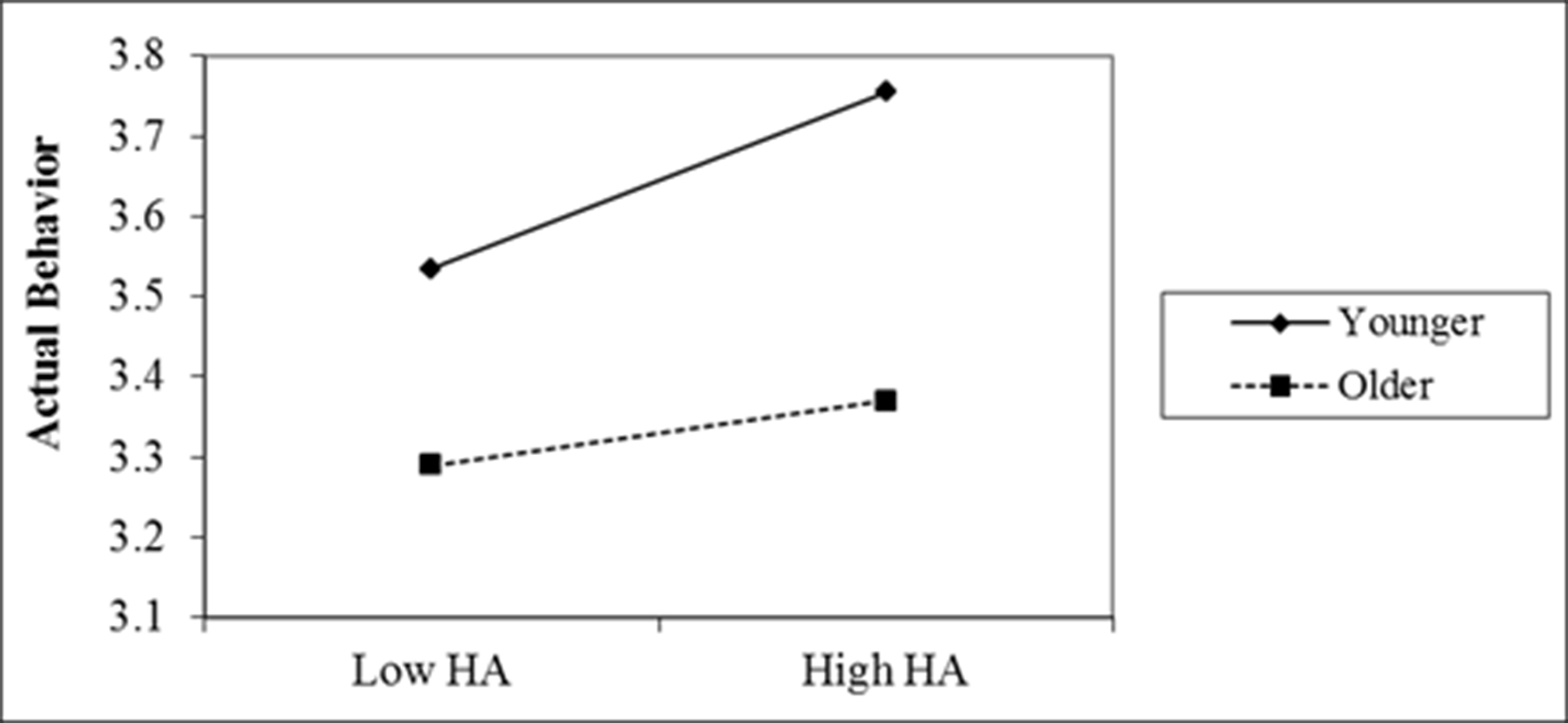

Supplement: S10 Fig — (TIF) [file pone.0210964.s010.tif]

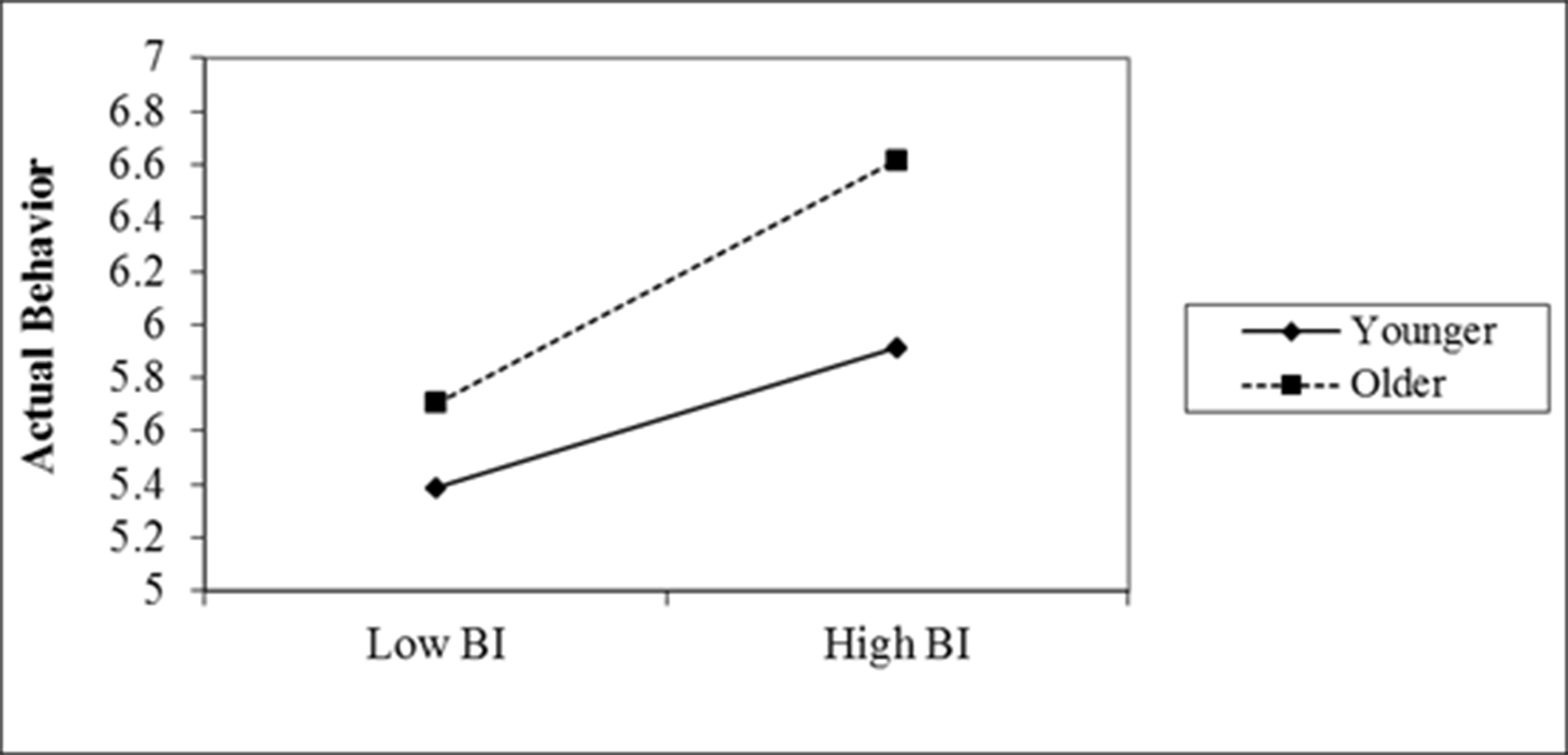

Supplement: S11 Fig — (TIF) [file pone.0210964.s011.tif]

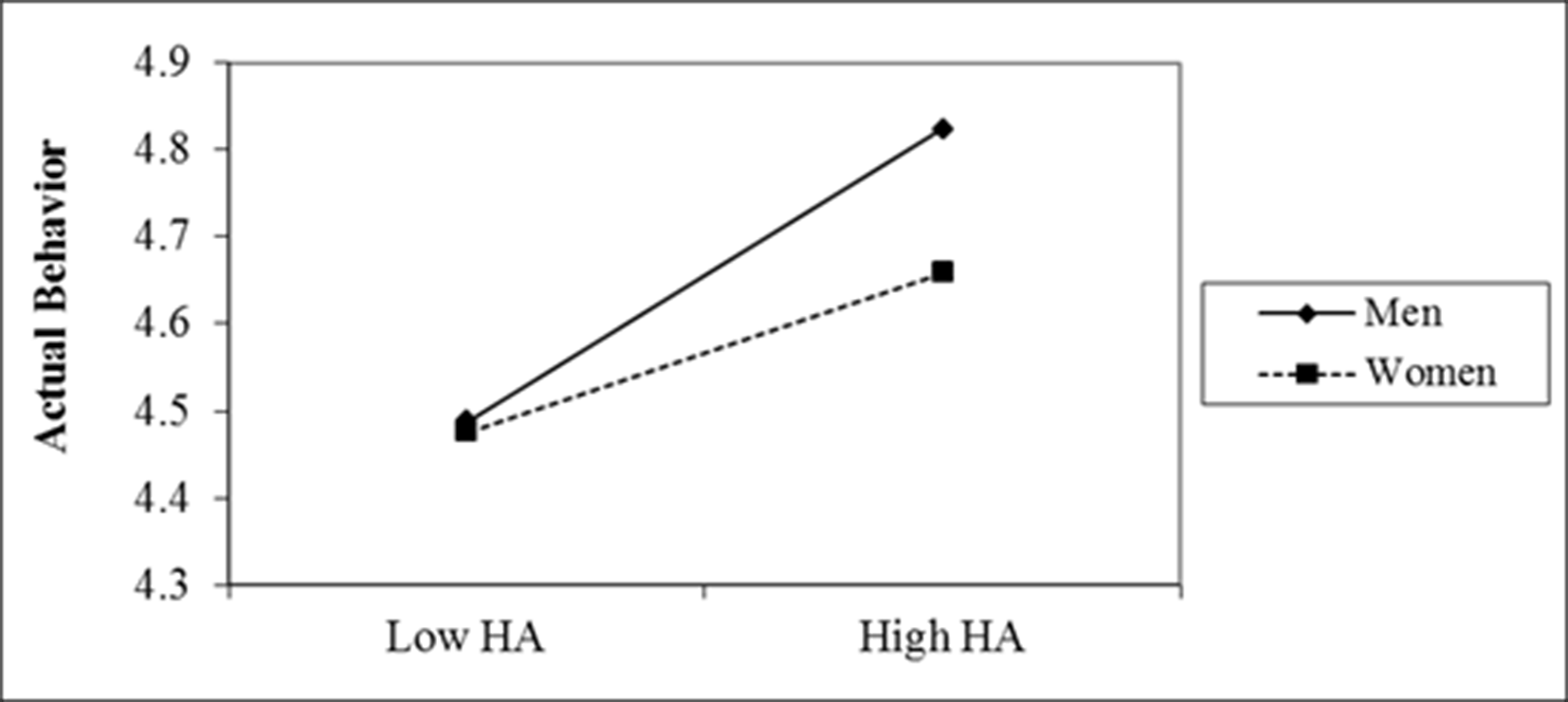

Supplement: S12 Fig — (TIF) [file pone.0210964.s012.tif]

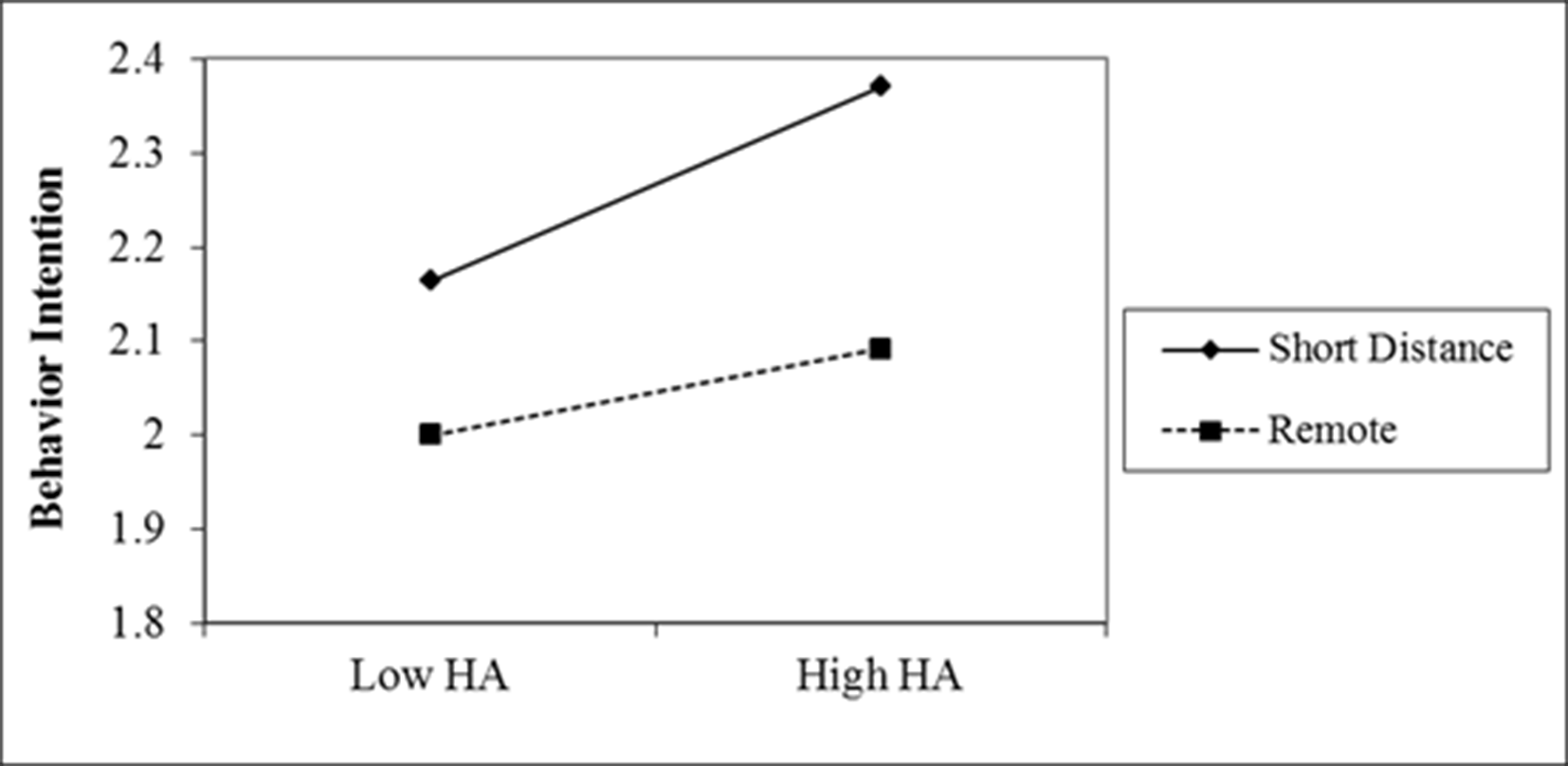

Supplement: S14 Fig — (TIF) [file pone.0210964.s014.tif]

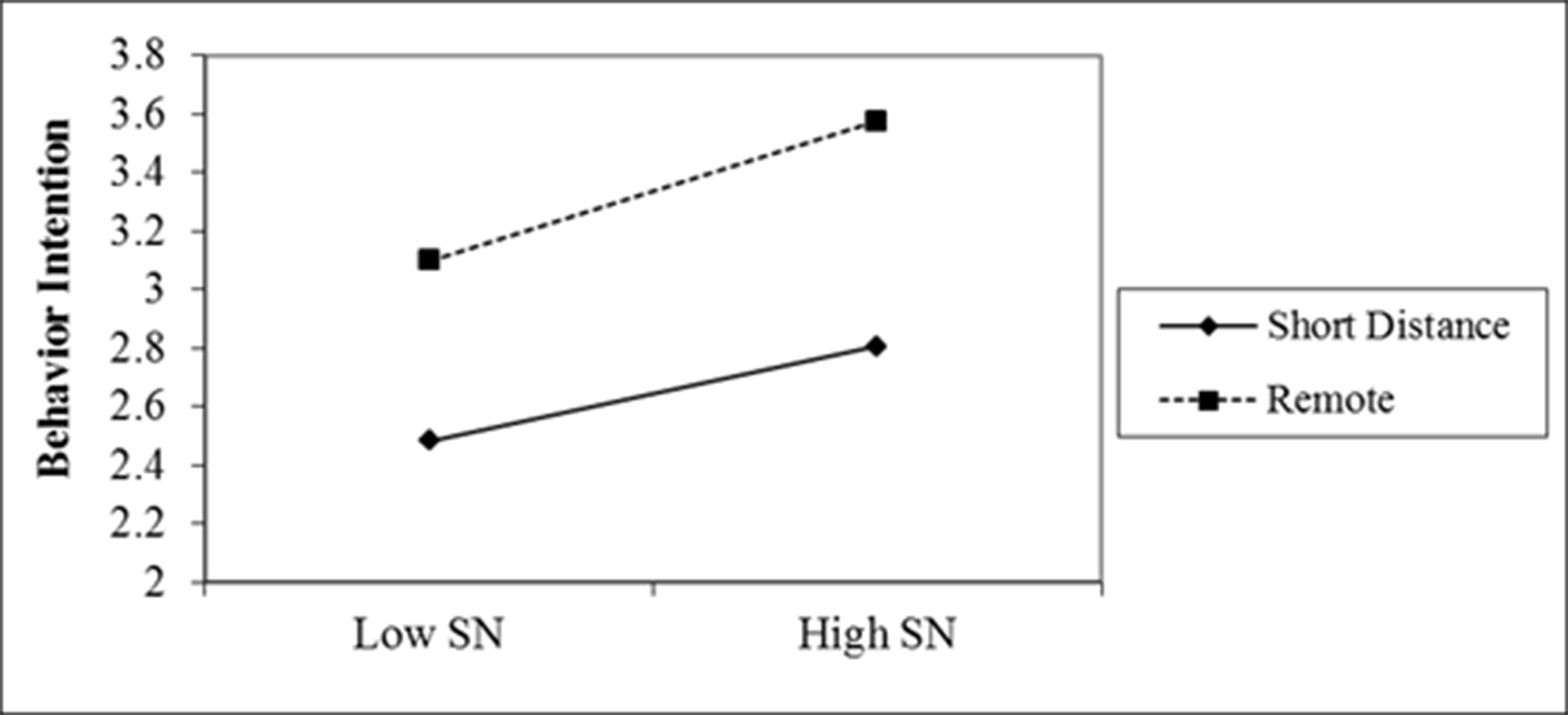

Supplement: S15 Fig — (TIF) [file pone.0210964.s015.tif]

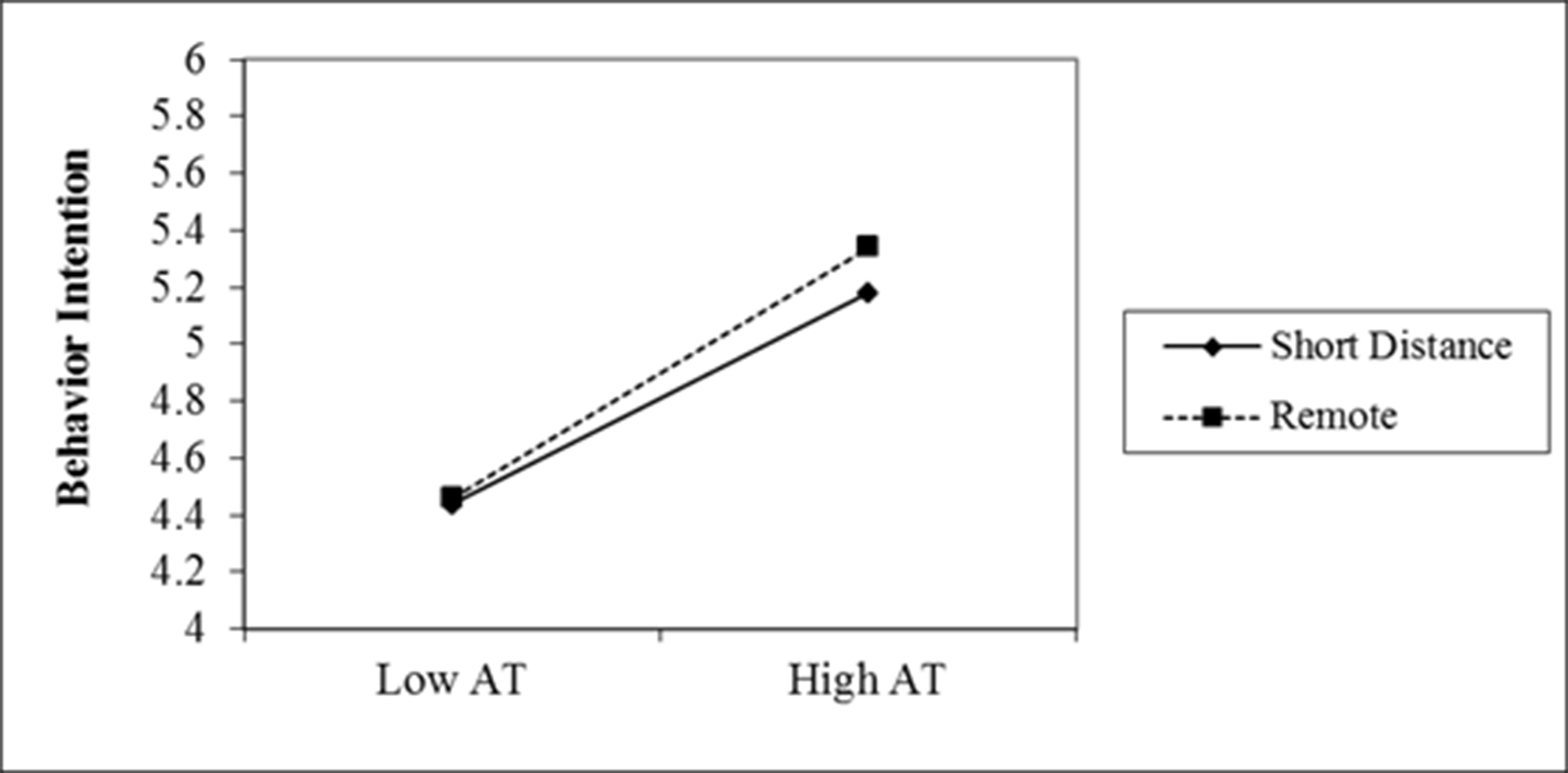

Supplement: S16 Fig — (TIF) [file pone.0210964.s016.tif]

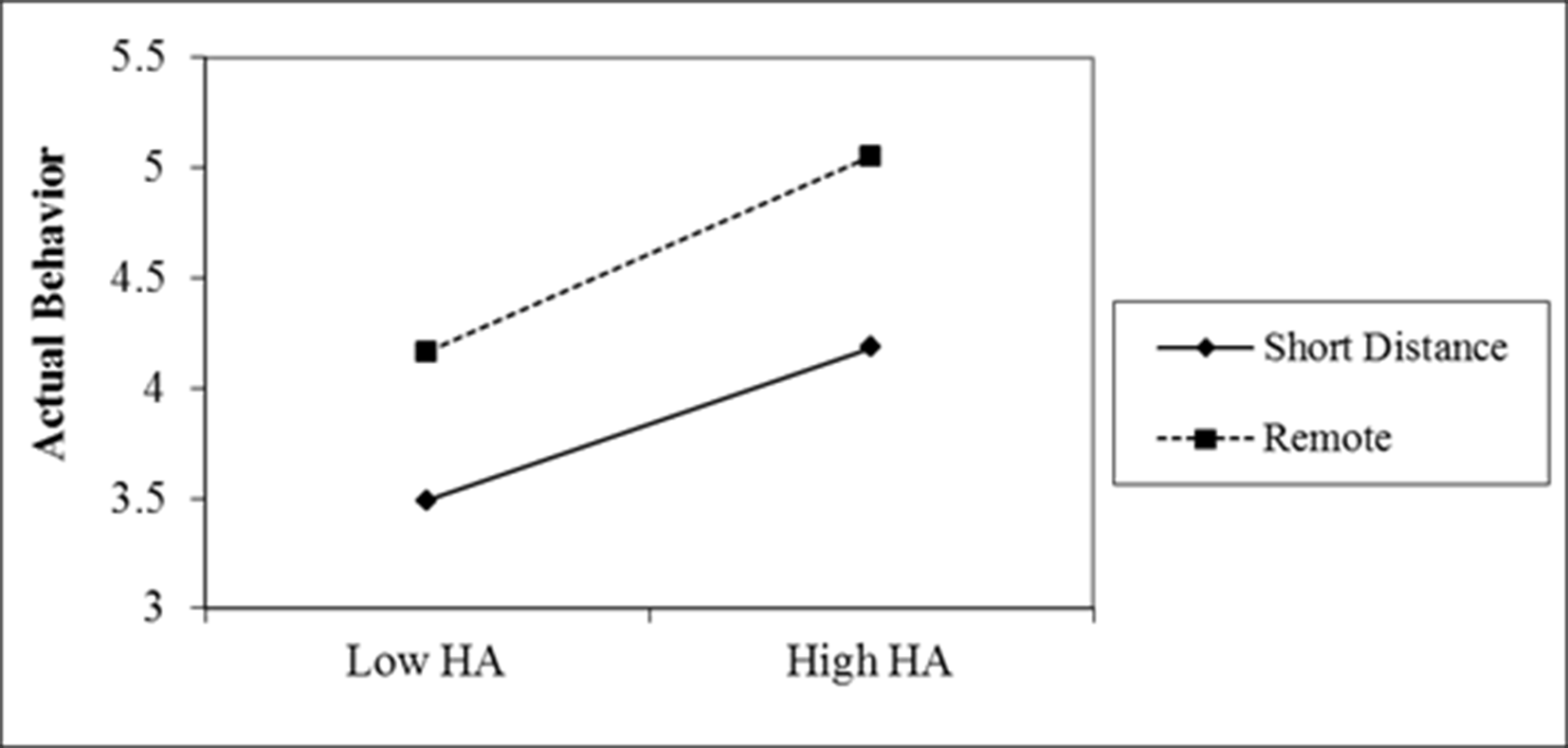

Supplement: S17 Fig — (TIF) [file pone.0210964.s017.tif]

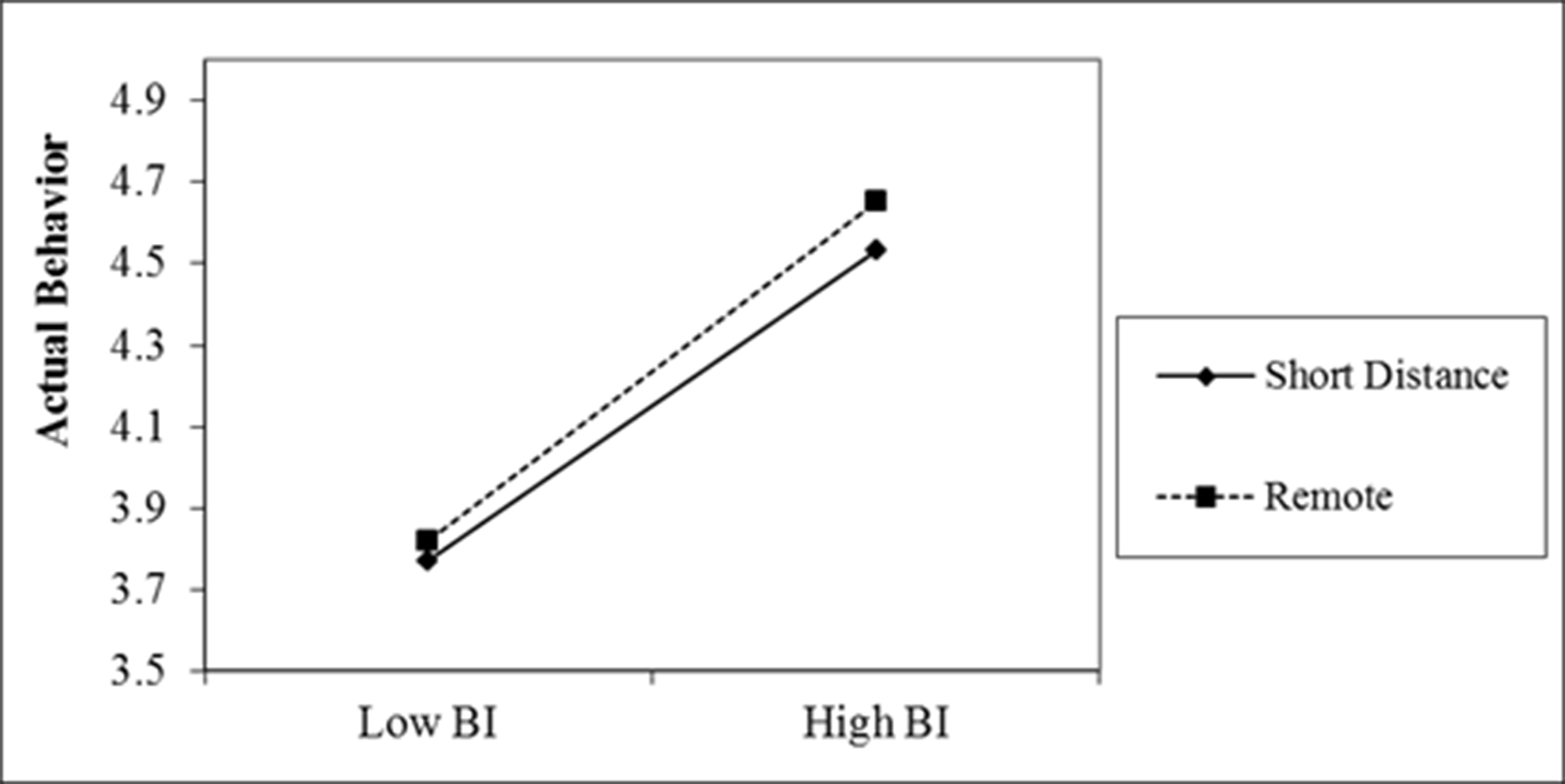

Supplement: S18 Fig — (TIF) [file pone.0210964.s018.tif]
